# Hepatic biomarker trajectories and mortality in septic adults with type 2 diabetes and recorded dipeptidyl peptidase-4 inhibitor exposure versus metformin-based therapy: A propensity-score-matched cohort study

**DOI:** 10.1016/j.metop.2026.100508

**Published:** 2026-10-10

**Authors:** Aristomenis G. Alevizopoulos, Georgios G. Anastasiou, Paraskevas Nomikos, Iakovos Kritikos

**Affiliations:** a Endocrine Unit and Diabetes Centre, Department of Clinical Therapeutics, Alexandra Hospital, School of Medicine, National and Kapodistrian University of Athens, Athens, Greece; b Third Department of Psychiatry, Agioi Anargyroi General Oncological Hospital of Kifisia, National and Kapodistrian University of Athens, Athens, Greece; c BIOSIM Laboratory, School of Electrical and Computer Engineering, National Technical University of Athens, Athens, Greece; d Department of Electrical and Electronic Engineering, University of West Attica, Thivon Avenue 250, Athens, 122 44, Greece; e Global Business School for Health, University College London, London, WC1E 6BT, UK

**Keywords:** Type 2 diabetes mellitus, DPP-4 inhibitors, Metformin, Sepsis, Liver injury, Pharmacoepidemiology

## Abstract

**Aims:**

To determine whether recorded dipeptidyl peptidase-4 (DPP-4) inhibitor exposure, compared with metformin, is associated with worse hepatic biomarker trajectories or mortality in adults with type 2 diabetes and sepsis.

**Methods:**

In three critical care databases (MIMIC-IV, eICU-CRD, NWICU), adults with type 2 diabetes and sepsis with a DPP-4 inhibitor record were matched 1:3 within each database to metformin comparators on age, sex, body mass index, 24-h SOFA and day-1 estimated glomerular filtration rate. The day 1–7 total bilirubin trajectory was analysed by linear mixed-effects regression under nine specifications including log transformation, flexible day terms, doubly robust adjustment and inverse-probability-of-observation weighting.

**Results:**

Matching yielded 105 DPP-4 inhibitor and 297 metformin patients; a pre-admission exposure record was present in 60.0%. The between-group difference in bilirubin slope was +0.048 mg/dL/day (95% CI −0.029 to 0.126; p = 0.221), with p between 0.109 and 0.436 across specifications. No other hepatic outcome survived multiplicity correction. Alkaline phosphatase rose more steeply on the primary specification (+6.97 U/L/day; q = 0.005) but was fragile: 12 of 28 sensitivity analyses retained p < 0.05. In-hospital mortality was 21.0% versus 13.5% (risk ratio 1.56, 95% CI 0.97–2.49), attenuating to 1.23 (0.67–2.29) with documented pre-admission exposure.

**Conclusions:**

There was no statistically detectable difference in bilirubin trajectory between recorded DPP-4 inhibitor and metformin exposure, although differences below the cohort's detectable-effect threshold cannot be excluded. An ALP signal was fragile and hypothesis-generating. Mortality findings are exploratory and non-causal. These associations with recorded exposure cannot inform inpatient management.

## Highlights

• Three ICU databases were pooled into one matched cohort of 402 septic T2D patients.
• No statistically detectable bilirubin difference: DPP-4 inhibitor vs metformin.
• This null held in all 26 sensitivity analyses of the primary outcome.
• A steeper ALP rise was fragile on robustness testing and is hypothesis-generating.
• Exploratory mortality excess attenuated with documented pre-admission exposure.

## 1 Introduction

Sepsis accounts for an estimated 11 million deaths annually, and the liver acts both as an effector of host defence and as a frequent target of injury [1], [2], [3]. Sepsis-associated liver dysfunction encompasses hypoxic hepatitis and intrahepatic cholestasis driven by inflammatory cytokines and downregulation of canalicular transporters; the resulting hyperbilirubinaemia is the hepatic component of the Sequential Organ Failure Assessment (SOFA) score and independently predicts worse outcome [3], [4].

Type 2 diabetes mellitus (T2DM) is highly prevalent among patients admitted with sepsis and modifies both susceptibility to infection and the metabolic response to it [5]. Chronic glucose-lowering therapy therefore constitutes a population-scale metabolic pre-exposure that adults with T2DM carry into acute critical illness, and whether it measurably alters the acute hepatic host response to sepsis is a question of direct relevance to metabolism research. Metformin and dipeptidyl peptidase-4 (DPP-4) inhibitors are the two oral agents most frequently recorded as background therapy in this population, yet their comparative hepatic safety in the setting of acute sepsis has almost no direct evidence base. The present study is observational and compares recorded medication exposure between arms. It does not compare continuation with discontinuation of therapy after admission and therefore cannot inform the inpatient management of these agents.

Both classes have anti-inflammatory properties in preclinical models: metformin principally through AMP-activated protein kinase signalling and suppression of nuclear factor-κB, and DPP-4 inhibitors through attenuation of reactive oxygen species generation, favourable Kupffer-cell polarisation and NF-κB inhibition [6], [7]. The hepatic effects of incretin signalling have been demonstrated most clearly in metabolic dysfunction-associated steatotic liver disease, where they are chronic and disease-modifying rather than acute [8], [9]. Whether either agent measurably alters the acute liver injury of human sepsis is unknown, and the critical-care literature is not consistent: large pharmacoepidemiologic cohorts suggest lower sepsis- and infection-related mortality among glucagon-like peptide-1 receptor agonist (GLP-1 RA) users than among DPP-4 inhibitor users, whereas elevated endogenous GLP-1 at intensive-care admission marks gut-barrier failure and predicts increased mortality [10], [11].

We therefore compared hepatic biomarker trajectories and mortality between septic adults with T2DM with a recorded DPP-4 inhibitor exposure and an active comparator of metformin-treated patients, using a propensity-score-matched design harmonised across three publicly available critical care databases. We hypothesised that hepatic biomarker trajectories over hospital days 1–7 would not differ between arms. The estimand is a DPP-4 inhibitor recorded in addition to background therapy versus metformin-based therapy without a DPP-4 inhibitor, not a comparison of mutually exclusive drug regimens.

## 2 Materials and methods

### 2.1 Data sources and study population

We pooled three de-identified critical care databases: MIMIC-IV v3.1 (Beth Israel Deaconess Medical Center, admissions 2008–2022), eICU-CRD v2.0 (over 200 United States hospitals, admissions 2014–2015) and NWICU v0.1.0 (Northwestern Memorial Hospital, admissions 2013–2023) [12], [13], [14]. Access was granted through PhysioNet following the mandatory training (CITI ID 76596039, A.G.A.). All data are de-identified, and individual consent was not required. Reporting follows the STROBE statement [15]. The three sources differ in period, case mix, medication-recording system, and severity-score availability, and span more than a decade over which sepsis management and antidiabetic prescribing both changed substantially. The source database was therefore retained as a covariate in the outcome models. eICU-CRD releases no admission year, and MIMIC-IV and NWICU shift each patient's dates by a patient-specific offset, so calendar time is represented through the source-database term. Shifted admission years were comparable between arms within MIMIC-IV and NWICU (Supplementary Table S3e). Database-specific estimates together with a leave-one-database-out analysis and formal heterogeneity assessment are reported.

Eligible patients were adults aged 18 years or older with type 2 diabetes and sepsis recorded during the index admission. Both conditions were identified from diagnosis codes or structured diagnosis entries; clinical Sepsis-3 criteria [16] were not applied. In MIMIC-IV and NWICU, which share the same table structure and were processed by the same query, sepsis was defined by ICD-9-CM 995.91, 995.92 or 785.52, or ICD-10-CM A41.x, R65.20, R65.21, A02.1, A22.7, A26.7, A32.7 or A42.7, and type 2 diabetes by ICD-9-CM 250.x0 or 250.x2, or ICD-10-CM E11.x, both recorded for the same hospital admission; these are discharge diagnoses and carry no onset time. In eICU-CRD, sepsis required a diagnosis entry for sepsis, severe sepsis, septic shock or septicaemia recorded within 24 h of unit admission, or an APACHE admission diagnosis of sepsis; type 2 diabetes required a diagnosis entry for diabetes mellitus or type 2 diabetes not indicating type 1 or insulin-dependent diabetes, or an associated ICD code of 250.x or E11.x. In MIMIC-IV and NWICU only the first qualifying admission per patient was retained; in eICU-CRD only the first unit stay of each hospital admission lasting at least 24 h. Complete code and string lists are given in Supplementary Table S0a and the codes that qualified the matched patients in Supplementary Table S0b; repeating the MIMIC-IV and NWICU queries with formatting-tolerant code matching identified no additional patients (Supplementary Table S0c). Each source-specific query emitted an identical canonical schema, so all downstream analysis was executed once on harmonised data. Screening counts by database are given in the Supplementary Material.

A companion analysis using the same cohort-assembly infrastructure, comparing incretin-based therapy (GLP-1 receptor agonists and DPP-4 inhibitors considered together) with non-incretin antidiabetic therapy in adults with type 2 diabetes and sepsis, was prepared separately; it was submitted to another journal, desk-rejected, and is not currently under consideration elsewhere. The two analyses differ in research question, exposure contrast (a heterogeneous incretin grouping versus a single active-comparator pair), comparator design, study population (the companion analysis draws on a single database, the present analysis on three) and outcome set. Overlap between them is confined to the description of the source databases and the cohort-assembly code; no patient-level result, table, or figure is shared.

### 2.2 Exposure and comparator definitions

Exposure was a recorded DPP-4 inhibitor order. Ascertainment differed by source. In MIMIC-IV and NWICU, orders were drawn from the prescriptions table by case-insensitive regex on generic and brand names covering sitagliptin, saxagliptin, linagliptin, alogliptin, and vildagliptin plus fixed-dose combinations, with the prescription start time as the timing variable. In eICU-CRD, exposure was drawn from the admissiondrug table, which holds the medication-reconciliation list compiled at unit admission and records therapy reported as being taken before arrival; drugoffset (minutes from unit admission) was retained for every record. In the exposed arm (n = 105), the DPP-4 inhibitors present were sitagliptin (n = 87, 82.9%), linagliptin (n = 14, 13.3%) and saxagliptin (n = 4, 3.8%); the arm is overwhelmingly sitagliptin- and linagliptin-based, and the saxagliptin subgroup is too small (n = 4) to drive the between-arm heart-failure or mortality signals. No source provides an outpatient dispensing record, so duration, adherence, and time since last dose are unknown throughout.

The comparator arm comprised patients with a metformin record and no DPP-4 inhibitor record. Fixed-dose DPP-4 inhibitor–metformin combinations were classified as DPP-4 inhibitor exposure, so the estimand is a DPP-4 inhibitor added to background therapy versus metformin-based therapy without a DPP-4 inhibitor, rather than a mutually exclusive drug comparison; 42.9% of the DPP-4 inhibitor arm was metformin-positive by construction. It is described as such in the title, abstract, Methods, and Conclusions. Two sensitivity analyses address the overlap directly: DPP-4 inhibitor plus metformin (n = 45) versus metformin alone, and DPP-4 inhibitor without a metformin record (n = 60) versus metformin alone. Concomitant sulfonylurea (43.8% versus 27.6%) and insulin use were carried into the doubly robust outcome models. Patients with any GLP-1 RA record were excluded from both arms, as there were too few to form a separate exposure category.

Time zero was defined as the hour of intensive-care admission for the index stay, and all baseline covariates were taken from the 24 h beginning at time zero. Study days 1–7 are indexed from time zero throughout. In addition, the terms “hospital day” and “ICU day” are not used interchangeably. Exposure status was assigned from medication records without reference to information arising after the first outcome measurement, so that exposure assignment does not use future information.

In the primary analysis, exposure was not restricted to the pre-admission period and therefore includes orders first written during the index admission, a permissive definition that admits misclassification. An audit of the exposure records showed that 63 of 105 (60.0%) exposed patients had a DPP-4 inhibitor record documented before admission: all 59 eICU-CRD patients, whose exposure is drawn from the admission medication-reconciliation list (entered a median of 0.7 h after unit admission), and 2 of 35 MIMIC-IV and 2 of 11 NWICU patients with a prescription start time before hospital admission. The remaining 42 patients had a first record written during the index admission. Because reconciliation entries are logged after arrival, 57 exposed patients (54.3%), 34 of them eICU-CRD reconciliation entries, have a first record timestamped after the first total-bilirubin measurement (Supplementary Table S2b and S2c). A sensitivity analysis restricts exposure to records documented before admission (61 exposed patients contributing trajectory data, compared with the full comparator arm) and is reported for the primary and ALP outcomes in Section 3.2. Stays without an admission-medication entry were dropped rather than coded unexposed.

### 2.3 Outcomes

The primary outcome was the day 1–7 trajectory of total bilirubin, selected because it is the hepatic component of the SOFA score and the analyte least susceptible to extrahepatic contamination. Secondary trajectory outcomes were alkaline phosphatase (ALP), aspartate aminotransferase, alanine aminotransferase, international normalised ratio (INR), albumin and platelet count. Composite indices (Model for End-Stage Liver Disease–Sodium [MELD-Na], Fibrosis-4 index, hepatic SOFA subscore) were examined as exploratory severity descriptors and are not interpreted as hepatic outcomes. Clinical outcomes were peak transaminases, nadir platelet count and all-cause mortality (in-hospital; 30- and 90-day where ascertainable).

The day 1–7 total bilirubin trajectory is the single confirmatory outcome. ALP was carried alongside bilirubin in the two-analyte false-discovery-rate family on the grounds that bilirubin indexes the hepatocellular and excretory axis while ALP indexes the cholestatic axis, these being the two hepatic patterns in which a class effect was considered biologically plausible. No dated statistical analysis plan preceded the analysis. The primary outcome and the two-analyte family were specified before models were fitted, but this cannot be independently verified. All other trajectory outcomes, all composite indices, and all mortality outcomes are exploratory and reported without inferential claim. A sensitivity analysis applying Benjamini–Hochberg correction across the full set of tested trajectory outcomes is reported in the Supplementary Material.

### 2.4 Propensity-score matching

DPP-4 inhibitor patients were matched 1:3 to metformin comparators by nearest-neighbour matching without replacement on the logit of the propensity score, within a caliper of 0.25 standard deviations, using age, body mass index, male sex, 24-h SOFA score and day-1 estimated glomerular filtration rate (eGFR; CKD-EPI 2021) [17]. The eGFR was included because metformin is contraindicated below 30 mL/min/1.73 m^2^, whereas DPP-4 inhibitors are not. Matching was within source because severity scoring is not comparable across datasets: MIMIC-IV supports a six-component SOFA whereas eICU-CRD permits only a reduced reconstruction. Balance was assessed by standardised mean difference (SMD), with |SMD|<0.10 taken as balanced and reported for every covariate before and after matching in a Love plot (Supplementary Fig. S1). Balance is a property of the matched sample rather than of a source population, so hypothesis tests are reported descriptively only and inference about balance rests on the SMD.

With 116 exposed patients, the number of covariates a propensity model can support is limited, so residual confounding was addressed by doubly robust estimation rather than by expanding the propensity model. Outcome models additionally adjust for source database, chronic liver disease, heart failure, sulfonylurea and insulin co-exposure, and day-1 values of albumin, sodium and the outcome analyte itself. Diabetes duration, glycated haemoglobin, statin and renin–angiotensin–system inhibitor use, and pre-sepsis glycaemic control are not recorded in any of the three databases and cannot be adjusted for. Their likely influence is quantified by E-value analyses (Section 2.5) rather than assumed away.

Neither day-1 eGFR nor 24-h SOFA is unambiguously a baseline covariate. Both are measured after the exposure of interest was established and both partly reflect sepsis-related organ injury, so conditioning on them risks overadjustment and, where they lie on a path between severity and outcome, collider bias. The primary specification retains them for comparability with prior critical-care pharmacoepidemiology. Two sensitivity analyses omit day-1 eGFR, and omit both day-1 eGFR and the 24-h SOFA score, from the propensity model, and are reported in Supplementary Tables S4, S4a and S4c.

### 2.5 Statistical analysis

Continuous variables are summarised as mean ± standard deviation and compared with the *t*-test or Mann–Whitney *U* test as appropriate; categorical variables are compared with the chi-square or Fisher exact test. Trajectories were modelled by linear mixed-effects regression (analyte ∼ group × day, random intercept per patient) with the metformin arm as the reference level. The group coefficient estimates the between-arm difference at baseline, the day coefficient the daily slope within the metformin arm, and the group-by-day interaction the divergence in daily slope, which was the term of primary interest. The Benjamini–Hochberg procedure was applied to the interaction p-values within a two-analyte family (total bilirubin, ALP); all other models are uncorrected and exploratory [18].

Total bilirubin, ALP, AST and ALT are markedly right-skewed. Each was therefore re-analysed on the natural-log scale as log(x + 1), with untransformed estimates retained for interpretability. To test whether the primary and ALP contrasts depended on model form, each was refitted under eight further specifications: (i) log transformation with linear day, (ii) raw scale with a random slope per patient for day, (iii) log with random slope, (iv) log with day entered as a categorical factor (joint Wald test across six interaction terms), (v) log with a restricted cubic spline on day (joint Wald over three interaction terms), (vi) log with doubly robust adjustment as in Section 2.4, (vii) log with additional adjustment for each analyte's own day-1 value, and (viii) log with source database added as a covariate. A within-patient change-from-day-1 mixed model was also specified but returned a singular covariance matrix; specification (vii), adjustment for each analyte's own day-1 value, addresses the same question. Models were fitted by restricted maximum likelihood.

Laboratory measurement across days 1–7 is not uniform: patients die, are discharged, or cease undergoing daily testing, and each of these is associated with clinical status. In the primary and ALP analyses, the number of patients contributing an ALP measurement fell from 60 (57.1%) on day 1 to 11 (10.5%) on day 7 in the exposed arm, and from 174 (58.6%) to 37 (12.5%) in the comparator arm. Daily denominators for every analyte, arm, and day are provided in Supplementary Table S6 and Fig. S6. Mixed-effects models use all available observations and give valid inference under a missing-at-random assumption conditional on the modelled covariates and each patient's observed prior measurements. This assumption is not testable and is unlikely to hold exactly. Four sensitivity analyses were therefore performed: inverse-probability-of-observation weighting, with the probability of contributing a measurement on each day modelled from treatment group, day, source database, day-1 severity and the preceding measured value; restriction to patients with at least three measurements of the analyte concerned; restriction to survivors to discharge; and truncation of follow-up at day 4, before denominators fall materially.

Mortality was compared by Fisher's exact test with risk ratios and 95% confidence intervals and, accounting for the matched design, by conditional logistic regression stratified on the matched set and by Poisson regression with generalised estimating equations clustered on the matched set. A model additionally adjusting for all 20 residually imbalanced covariates was inspected for numerical stability and not interpreted because it produced complete separation (Section 3.3). Between-database heterogeneity in the primary and ALP contrasts was assessed by fitting database-specific estimates, combining them under a DerSimonian–Laird random-effects model with I^2^ and Cochran Q, and by a leave-one-database-out analysis.

No dated statistical analysis plan preceded the study, and a formal a priori sample-size calculation cannot be constructed retrospectively. The minimum detectable effect at 80% power and α = 0.05 was therefore computed for the primary trajectory contrast and for in-hospital mortality (Section 3.1). The robustness of any positive finding to unmeasured confounding was quantified by the E-value, the minimum association on the risk-ratio scale that an unmeasured confounder would need to have with both exposure and outcome, conditional on measured covariates, to account for the observed estimate.

Two-sided p < 0.05, or q < 0.05, was considered significant. Analyses used Python (pandas, statsmodels, lifelines) and R (lme4, survival). A large language model assisted with code refactoring and language editing only; all code and text were verified by the authors.

## 3 Results

### 3.1 Cohort and balance

Of 1623 eligible patients (116 DPP-4 inhibitor, 1507 metformin), matching retained 105 of 116 exposed patients and selected 297 comparators, yielding a matched cohort of 402 (Fig. 1). eICU-CRD contributed 55.7% of the matched cohort, MIMIC-IV 34.8%, and NWICU 9.5%. Mean age was 71.5 years in both arms. At 80% power and α = 0.05, this cohort could detect a between-arm difference in in-hospital mortality of 12.3 percentage points (risk ratio 1.91) and a group-by-day difference in total bilirubin of 0.11 mg/dL/day. Differences smaller than these are not resolvable in this sample, and both the observed 7.5-percentage-point mortality difference and the null trajectory findings should be read against that limit rather than as evidence of no effect.

**Fig. 1:**
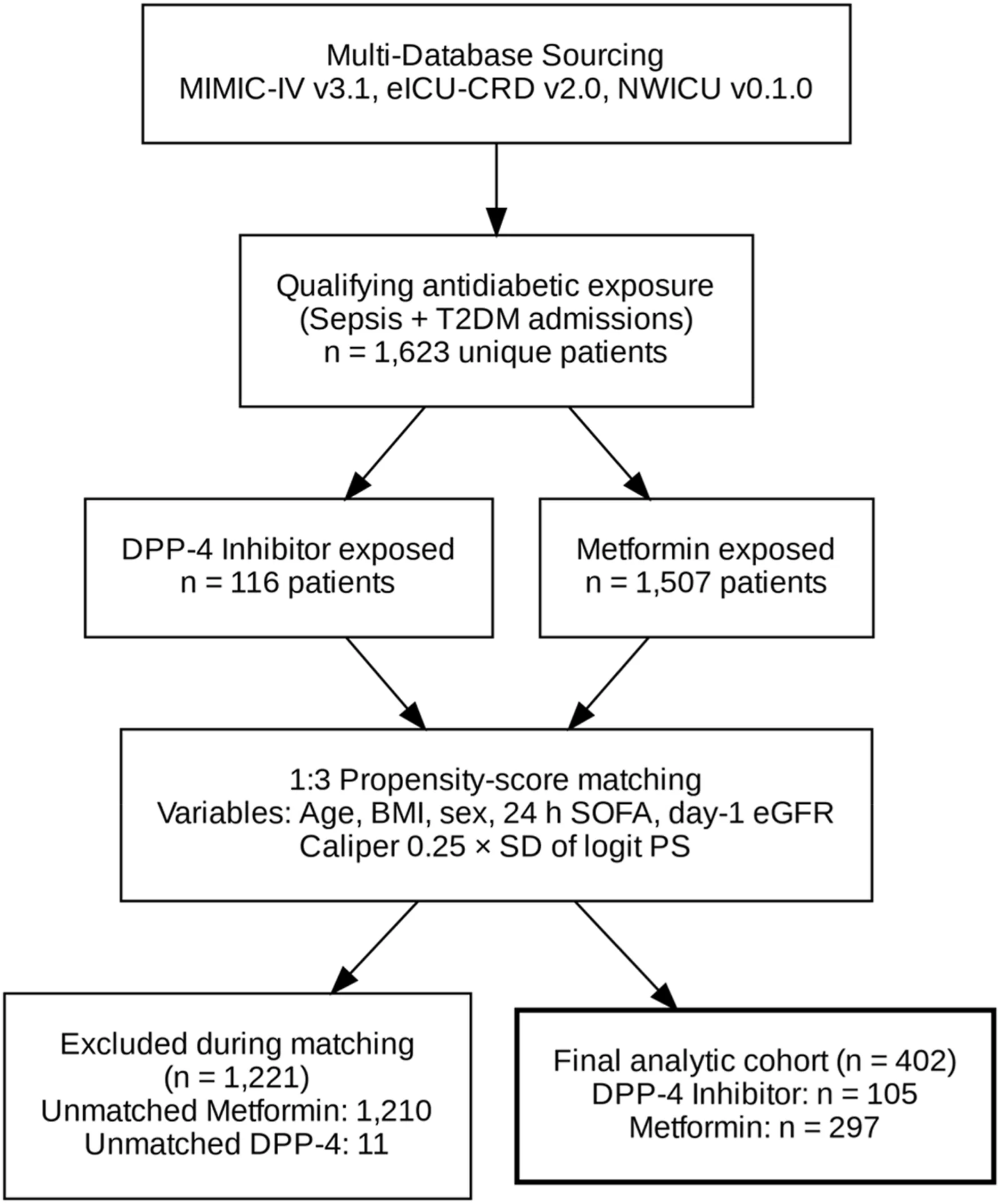
Participant selection flowchart.

A per-patient audit of the exposure records was performed (Supplementary Table S2a–S2d). Sixty-three of 105 exposed patients (60.0%) had a DPP-4 inhibitor record documented before admission: 59 of 59 in eICU-CRD from admission medication-reconciliation entries (median 0.7 h after unit admission, IQR 0.3–1.8), and 2 of 35 in MIMIC-IV and 2 of 11 in NWICU from prescriptions written before hospital admission. The remaining 42 (MIMIC-IV 33, NWICU 9) had a first record written during the index admission. Fifty-seven exposed patients (54.3%) had a first record timestamped after the first total-bilirubin measurement; 34 of these were eICU-CRD reconciliation entries, which record therapy taken before arrival but are logged shortly after it. Recorded exposure therefore cannot be equated with uninterrupted pre-admission therapy in every patient.

Balance on the five matched covariates was excellent, with all standardised mean differences below 0.05 (Table 1; Supplementary Fig. S1). Residual imbalance persisted for variables outside the propensity model: albumin (2.97 ± 0.67 vs 2.76 ± 0.61 g/dL; SMD 0.343), sodium (SMD 0.246), fibrinogen (−0.277) and activated partial thromboplastin time (−0.235). Day-1 ALP was modestly higher in the exposed arm (127.2 ± 97.9 vs 117.4 ± 93.4 U/L; SMD 0.103). Sulfonylurea co-exposure was more common in the DPP-4 inhibitor arm (43.8% vs 27.6%). Day-1 total bilirubin was lower in the exposed arm (SMD −0.213), chronic liver disease was more common (SMD 0.153), and heart failure was more common (SMD 0.148). Every variable with |SMD| above 0.10 was carried into the doubly robust outcome models described in Section 2.4.

**Table 1:** Baseline characteristics of the DPP-4 inhibitor and metformin cohorts after 1:3 propensity score matching.

Characteristic	DPP-4 (n = 105)	METFORMIN (n = 297)	SMD	P-value
Age, years	71.52 ± 13.13	71.47 ± 12.93	0.004	0.970
Sex				
Male	57 (54.3)	163 (54.9)	−0.012	1.000
Female	48 (45.7)	134 (45.1)	—	—
Race				
Asian	4 (3.8)	12 (4.0)	—	0.214
Black	10 (9.5)	40 (13.5)	—	—
Hispanic	3 (2.9)	21 (7.1)	—	—
Other/Unknown	17 (16.2)	30 (10.1)	—	—
White	71 (67.6)	194 (65.3)	—	—
Body mass index, kg/m^2^	31.39 ± 8.52	31.20 ± 9.25	0.021	0.861
SOFA score (24 h)	2.87 ± 3.06	2.95 ± 3.06	−0.028	0.804
***Renal parameters***				
eGFR, mL/min/1.73 m^2^	48.66 ± 29.67	49.91 ± 28.70	−0.043	0.727
Creatinine, mg/dL	1.85 ± 1.19	1.90 ± 1.59	−0.037	0.743
BUN, mg/dL	36.51 ± 25.89	37.07 ± 26.03	−0.022	0.859
***Routine biochemistry and inflammatory markers***				
Lactate, mmol/L	2.57 ± 1.92	2.65 ± 2.42	−0.038	0.797
Sodium (Na^+^), mmol/L	137.75 ± 5.17	136.40 ± 5.76	0.246	0.040
Potassium (K^+^), mmol/L	4.28 ± 0.67	4.24 ± 0.66	0.060	0.626
Albumin, g/dL	2.97 ± 0.67	2.76 ± 0.61	0.343	0.036
***Liver function tests and hepatic severity indices***				
Total bilirubin, mg/dL	0.87 ± 0.84	1.16 ± 1.74	−0.213	0.091
ALT, U/L	74.61 ± 185.70	70.20 ± 172.51	0.025	0.872
AST, U/L	155.43 ± 453.30	93.22 ± 255.41	0.170	0.317
ALP, U/L	127.20 ± 97.89	117.38 ± 93.40	0.103	0.500
INR	1.54 ± 0.72	1.66 ± 0.88	−0.143	0.328
Fibrinogen, mg/dL	435.25 ± 223.30	489.35 ± 190.13	−0.277	0.603
aPTT, s	34.37 ± 10.32	37.89 ± 18.68	−0.235	0.100
Platelets, ×10^9^/L	213.19 ± 114.87	215.24 ± 120.73	−0.017	0.886
MELD-Na	16.42 ± 7.30	18.26 ± 7.91	−0.244	0.165
Hepatic SOFA (bilirubin subscore)	0.27 ± 0.61	0.37 ± 0.81	−0.150	0.284
FIB-4	5.35 ± 8.40	5.27 ± 8.21	0.009	0.951
***Comorbidities***				
Heart failure	32 (30.5)	71 (23.9)	0.148	0.232
Coronary artery disease	30 (28.6)	72 (24.2)	0.098	0.456
COPD	13 (12.4)	40 (13.5)	−0.032	0.908
Chronic liver disease	8 (7.6)	12 (4.0)	0.153	0.235
***Baseline antidiabetic medications***				
DPP-4 inhibitor	105 (100.0)	0 (0.0)	—	—
Metformin	45 (42.9)	297 (100.0)	—	—
Sulfonylurea	46 (43.8)	82 (27.6)	—	—
Thiazolidinedione	8 (7.6)	7 (2.4)	—	—
SGLT2 inhibitor	2 (1.9)	1 (0.3)	—	—
Insulin (basal/intermediate)	23 (21.9)	69 (23.2)	—	—
Insulin (prandial/regular)	27 (25.7)	59 (19.9)	—	—
Insulin (any)	33 (31.4)	87 (29.3)	—	—

Values are shown for the propensity-score-matched cohort (DPP-4, n = 105; METFORMIN, n = 297; nearest-neighbour matching, ratio ∼1:3). Data are n (%) for categorical variables and mean ± SD for continuous variables. Per-analyte denominators, where a measurement was not available for every matched patient, are reported by analyte, arm and study day in the Supplementary Material rather than in this table. Balance was judged on |SMD| < 0.10; residual imbalance above that threshold persisted for albumin, sodium, fibrinogen, aPTT, day-1 total bilirubin, day-1 ALP, MELD-Na, chronic liver disease and heart failure, all of which are carried into the doubly robust outcome models (Section 2.4). P-values are shown for description only and are not the basis on which balance was judged. Standardised mean differences for all covariates before and after matching are shown as a Love plot in Supplementary Fig. S1.

### 3.2 Hepatic biomarker trajectories

The primary outcome was null. Total bilirubin trajectories did not diverge between arms (group-by-day interaction +0.048 mg/dL/day, 95% CI −0.029 to 0.126; p = 0.221). On the raw scale, aminotransferase, INR, albumin and platelet trajectories were likewise indistinguishable (Supplementary Table S8), and platelet counts recovered at a comparable rate in both arms (metformin slope +3.59 × 109/L/day, p < 0.001; interaction p = 0.742). Peak aspartate aminotransferase, peak alanine aminotransferase and nadir platelet count did not differ (Supplementary Table S10). The primary bilirubin null was robust. Twenty-six bilirubin estimates across all sensitivity analyses had a confidence interval including zero (lowest p = 0.086; Supplementary Table S8b and Fig. S8b). Across the nine model specifications, interaction p ranged from 0.109 to 0.436 across the raw-scale linear, log-linear, random-slope, categorical-day, spline, doubly robust, own-day-1-adjusted, and source-adjusted models, and under inverse-probability-of-observation weighting. Full specification-by-specification estimates are reported in Supplementary Table S8b. Daily numbers of contributing patients and measurements by analyte and arm are given in Supplementary Table S6, and fall materially across the week; estimates for later days therefore rest on a smaller and increasingly selected sample. On the log scale, AST (+0.061 log units/day, p = 0.043), ALT (+0.050, p = 0.047) and lactate (+0.294, p = 0.021) slopes were nominally steeper in the exposed arm, but none survived Benjamini–Hochberg correction across the full trajectory family (q = 0.095 for each; Supplementary Table S5b).

A between-arm difference in linear ALP slope emerged and is reported as hypothesis-generating. ALP rose more steeply in the DPP-4 inhibitor arm (group-by-day interaction +6.97 U/L/day, 95% CI 2.49 to 11.45; p = 0.002, q = 0.005) on the primary specification, corresponding to a cumulative separation of approximately 49 U/L over seven days, against a metformin arm whose ALP was essentially flat across the week (−0.68 U/L/day, p = 0.535). On formal robustness testing across 28 sensitivity analyses evaluated in the revised analysis, 12 retained p < 0.05. The signal survived log transformation of the analyte (+0.027 log U/L/day, 95% CI + 0.002 to +0.052; p = 0.036), adjustment for each patient's own day-1 ALP (+0.038, 95% CI + 0.012 to +0.064; p = 0.004), doubly robust adjustment for source database and residual imbalances (+0.041, 95% CI + 0.010 to +0.072; p = 0.009), and addition of source database as a covariate (+0.026, 95% CI + 0.002 to +0.051; p = 0.037). It did not survive allowing per-patient slopes for day, either on the raw scale (+3.33 U/L/day, 95% CI −3.82 to +10.47; p = 0.362) or on the log scale (+0.006, 95% CI −0.031 to +0.043; p = 0.745), nor relaxation of the linear day term (day as factor, joint Wald p = 0.341; restricted cubic spline, joint Wald p = 0.099). The effect is therefore located specifically in the linear day term of a random-intercept-only covariance structure, which is consistent both with a slow cumulative pharmacodynamic effect and with progressive informative dropout selecting for higher-ALP survivors, and the data do not distinguish these interpretations.

No single database reached statistical significance in isolation: MIMIC-IV (n = 105) yielded +0.043 (95% CI −0.001 to +0.088; p = 0.054); eICU-CRD (n = 190) +0.015 (−0.015 to +0.046; p = 0.328); NWICU contributed too few repeated measurements to converge. Formal heterogeneity was negligible for ALP (I^2^ 5.1%, Cochran Q p = 0.305) as for bilirubin (I^2^ 0%, Q p = 0.629; Supplementary Table S3c), and both fitting databases pointed in the same direction. However, leave-one-database-out estimates showed that statistical significance of the pooled estimate depended on the inclusion of MIMIC-IV: with MIMIC-IV omitted, the estimate was +0.015 (−0.015 to +0.046; p = 0.328). Under DerSimonian–Laird random-effects pooling, the estimate was +0.025 (−0.001 to +0.051), crossing the null, while the fixed-effect mixed model reached p = 0.036.

Excluding the 20 patients with coded chronic liver disease strengthened the ALP contrast (+0.033, 95% CI + 0.006 to +0.060; p = 0.016), and adjustment for concomitant sulfonylurea and insulin therapy left it unchanged (+0.027; p = 0.035). It was not retained when exposure was restricted to records documented before admission (+0.022, 95% CI −0.006 to +0.050; p = 0.123), in either stratum of the metformin co-exposure decomposition (p = 0.083 and p = 0.108), or when day-1 eGFR was omitted from the propensity model (+0.007; p = 0.630) (Supplementary Table S4 and S4b). Under inverse-probability-of-observation weighting the ALP point estimate was larger (+0.035) but less precise (95% CI −0.015 to +0.085; p = 0.166); restriction to patients with at least three ALP measurements retained significance (+0.038; p = 0.006), whereas restriction to survivors to discharge (+0.004; p = 0.769) or to days 1–4, when denominators are intact (+0.016; p = 0.491), did not (Supplementary Table S6c). The bilirubin null held in every one of these analyses. The day-7 denominator was 11 exposed and 37 comparator patients (89.5% and 87.5% missing respectively), and no interpretation of the ALP finding that relies on later-week estimates should be advanced without weight given to this. Among the exploratory composite indices, MELD-Na also diverged (+0.97 points/day, 95% CI 0.32 to 1.61; p = 0.003, uncorrected). Full model output is given in Table 2 and trajectories are shown in Fig. 2.

**Table 2:** Day 1–7 mixed-effects analysis of hepatic biomarker trajectories, DPP-4 inhibitor versus metformin.

Analyte	Group effect (DPP-4 vs METFORMIN) est (95% CI); p	Daily slope, METFORMIN est per day (95% CI); p	Group × Day interaction est per day (95% CI); p	FDR q	FDR sig.
Total bilirubin, mg/dL	−0.280 (−0.656, 0.096); p = 0.144	−0.055 (−0.092, −0.017); p = 0.004	+0.048 (−0.029, 0.126); p = 0.221	0.221	No
ALP, U/L	−6.981 (−30.912, 16.951); p = 0.568	−0.682 (−2.840, 1.475); p = 0.535	+6.969 (2.486, 11.452); p = 0.002	0.005	Yes
Platelets, ×10^9^/L	−5.053 (−32.052, 21.946); p = 0.714	+3.585 (2.295, 4.875); p < 0.001	+0.433 (−2.147, 3.014); p = 0.742	—	—
Albumin, g/dL	+0.146 (−0.024, 0.316); p = 0.093	−0.048 (−0.063, −0.034); p < 0.001	−0.015 (−0.043, 0.014); p = 0.323	—	—
MELD-Na	−1.915 (−4.484, 0.654); p = 0.144	−0.464 (−0.734, −0.195); p < 0.001	+0.966 (0.323, 1.609); p = 0.003	—	—
Hepatic SOFA (bilirubin subscore)	−0.045 (−0.237, 0.148); p = 0.648	+0.002 (−0.015, 0.019); p = 0.836	−0.009 (−0.044, 0.026); p = 0.632	—	—
FIB-4	−0.369 (−2.906, 2.168); p = 0.776	−0.276 (−0.559, 0.007); p = 0.056	+0.515 (−0.080, 1.110); p = 0.090	—	—

**Fig. 2:**
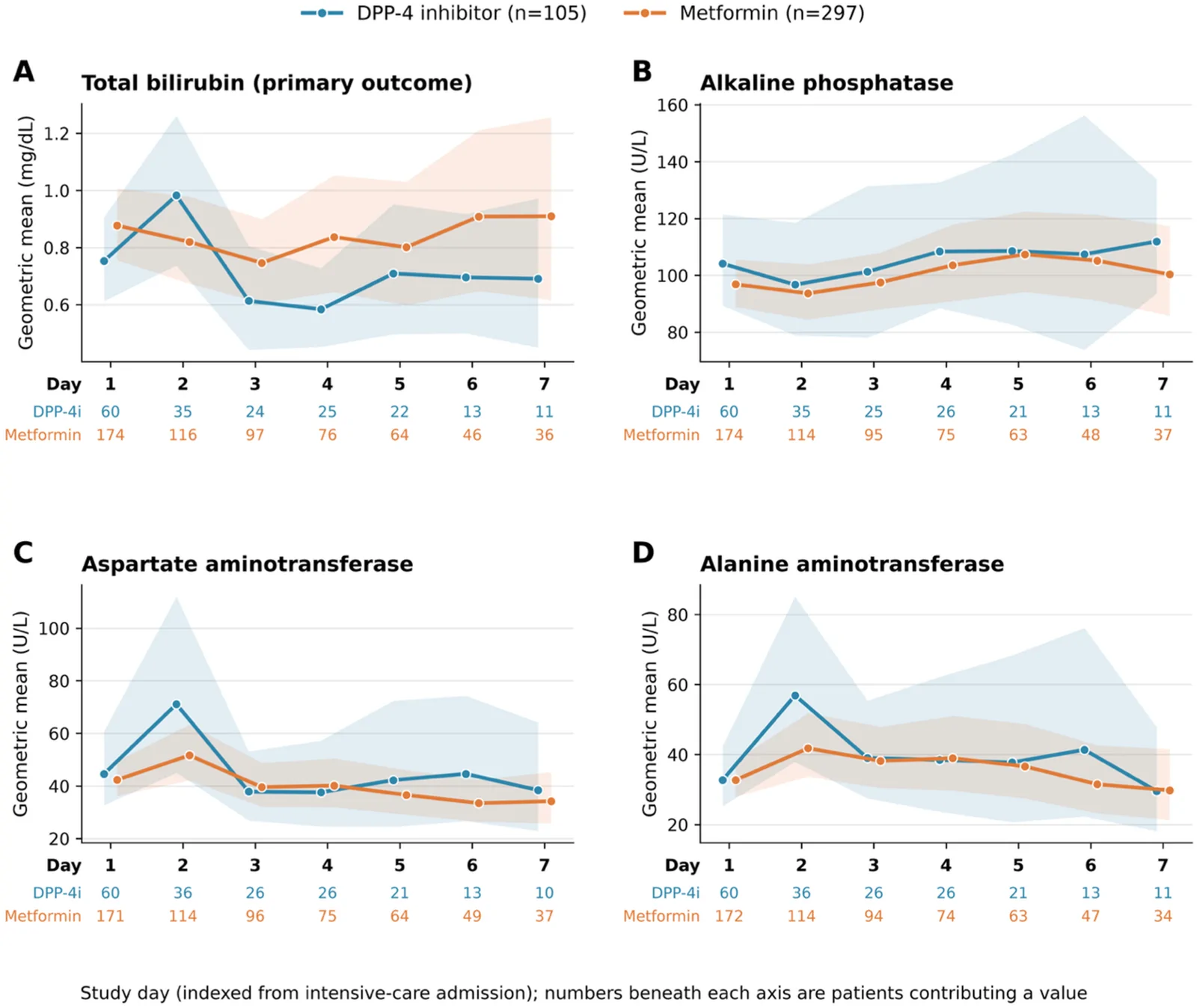
Daily trajectories of total bilirubin (A, primary outcome), alkaline phosphatase (B), aspartate aminotransferase (C) and alanine aminotransferase (D) over study days 1–7 in the matched cohort, by exposure arm. Points are geometric means (back-transformed mean of log[x+1]) with 95% confidence bands; arms are offset horizontally for legibility. Numbers beneath each panel are patients contributing a value on that day. Days with fewer than three contributing patients in an arm are not plotted. Trajectories of synthetic-function, coagulation, lactate, and composite indices are shown in Supplementary Fig. S9.

Linear mixed-effects models were fit separately for each hepatic analyte across days 1–7 of the hospital stay, indexed from time zero (the hour of intensive-care admission), in the propensity-score-matched cohort, with random intercepts per patient and fixed effects for treatment group (METFORMIN as reference), day, and their interaction. The Group effect column reports the estimated between-group difference at day 0; the Daily slope column reports the within-METFORMIN change per day; the Group × Day interaction column reports the difference in daily slope between DPP-4 and METFORMIN. AST, ALT, and INR are omitted here for length and reported with full model output in Supplementary Table S8; log-scale and additional specifications for total bilirubin and ALP are given in Supplementary Tables S5, S8a, and S8b.

### 3.3 Mortality

In-hospital mortality was 21.0% (22/105) with DPP-4 inhibitor exposure and 13.5% (40/297) with metformin (risk ratio 1.56, 95% CI 0.97 to 2.49; p = 0.083). Analyses accounting for the matched design were concordant: conditional logistic regression stratified on matched set gave an odds ratio of 1.74 (95% CI 0.96 to 3.17; p = 0.068), and Poisson regression with generalised estimating equations clustered on matched set a risk ratio of 1.55 (0.98 to 2.47; p = 0.063) (Supplementary Table S7b). A model additionally adjusting for all 20 residually imbalanced covariates produced complete separation against 62 events and was not interpreted. Restricted to the 63 exposed patients with documented pre-admission exposure and their own matched comparators, in-hospital mortality was 19.0% (12/63) versus 15.4% (27/175) (risk ratio 1.23, 95% CI 0.67 to 2.29; p = 0.553; conditional logistic odds ratio 1.26, 0.58 to 2.72). The crude excess was concentrated among the 42 patients whose exposure was first recorded during the admission (23.8% versus 13.5%; risk ratio 1.77, 0.96 to 3.26), 33 of whom were in MIMIC-IV (30.3% versus 13.3%; risk ratio 2.27, 1.12 to 4.63), and is therefore susceptible to exposure-timing bias (Supplementary Table S7e). An unmeasured confounder would need an association of at least 2.49 on the risk-ratio scale with both exposure and in-hospital mortality, conditional on measured covariates, to explain this point estimate away; because the lower confidence bound crosses the null, the corresponding E-value for the confidence limit is 1.00, and the association the E-value protects is not itself statistically significant. This is a point estimate consistent with harm that the cohort is underpowered to resolve rather than a demonstrated null, and the upper confidence bound does not exclude a clinically important excess. The minimum in-hospital mortality difference this cohort could detect at 80% power was 12.3 percentage points (Section 3.1); the observed 7.5-point difference is below that threshold, which is why p = 0.083 is unresolved rather than null. In-hospital mortality ascertainment was complete in all three databases (Supplementary Table S7c). All mortality analyses are exploratory and do not support a causal interpretation. Linked out-of-hospital death dates are available only in MIMIC-IV: eICU-CRD records none, and the completeness of the few post-discharge death dates in NWICU cannot be verified. Thirty- and 90-day mortality were therefore assessed in MIMIC-IV only (35 exposed, 105 comparators): 30-day mortality was 25.7% versus 16.2% (risk ratio 1.59, 95% CI 0.78 to 3.24; p = 0.218) and 90-day mortality 31.4% versus 25.7% (risk ratio 1.22, 95% CI 0.68 to 2.20; p = 0.517) (Supplementary Table S7a). MIMIC-IV patients were older and had higher in-hospital mortality than those in the other databases (Supplementary Table S3), so these estimates are not representative of the matched cohort. In Cox analysis of 90-day survival in MIMIC-IV, the hazard ratio for DPP-4 inhibitor exposure was 1.36 (95% CI 0.67 to 2.73; p = 0.394). The 90-day Kaplan-Meier survival graph is illustrated in Fig. 3.

**Fig. 3:**
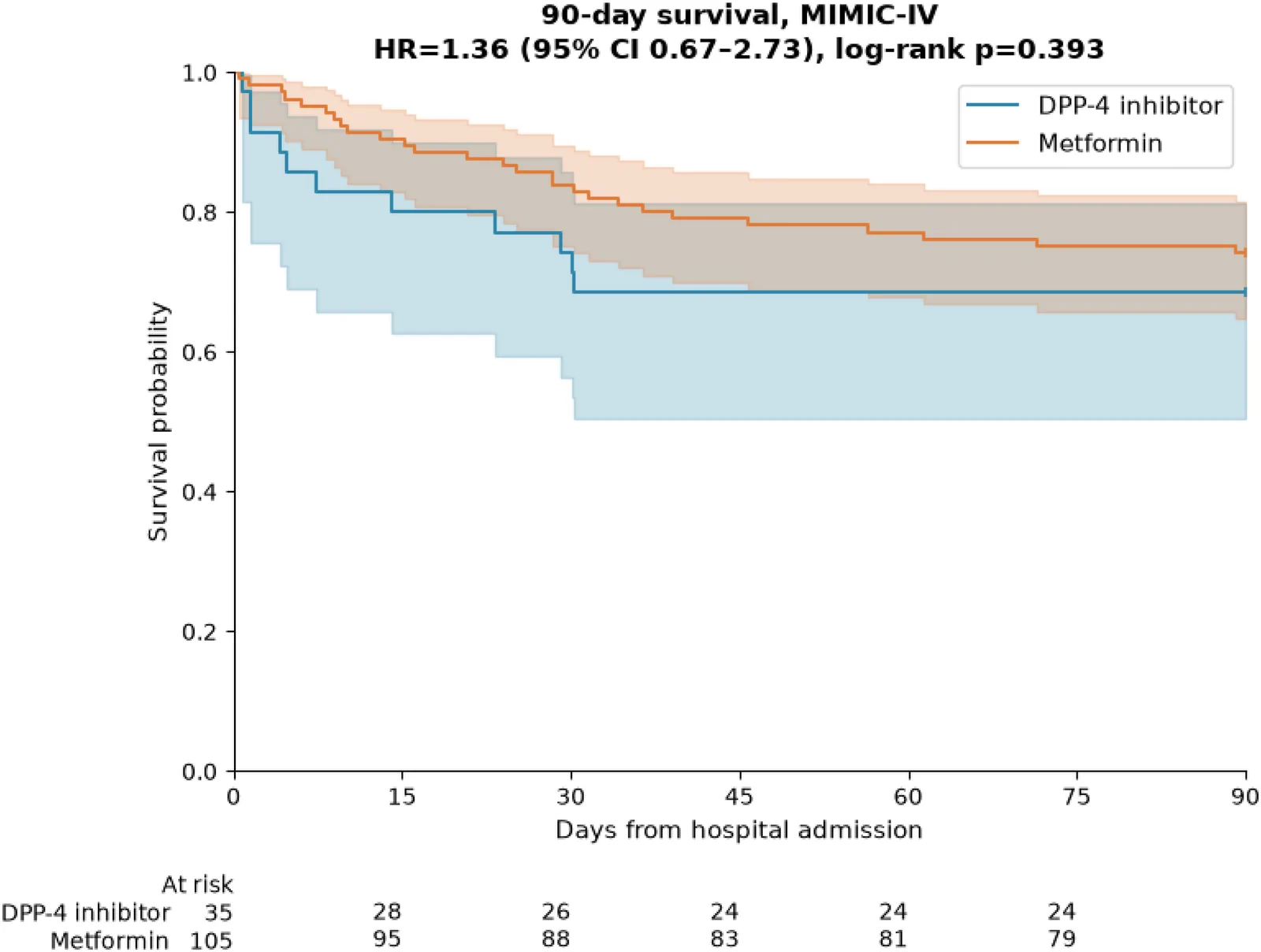
Kaplan–Meier estimates of 90-day survival by exposure arm in MIMIC-IV (35 DPP-4 inhibitor and 105 metformin patients), with 95% confidence bands.

## 4 Discussion

In a propensity-score-matched cohort of 402 septic adults with T2DM assembled from three critical care databases, there was no statistically detectable difference between recorded DPP-4 inhibitor and metformin exposure in bilirubin, aminotransferase, INR, albumin or platelet trajectories, or in peak transaminases. The principal contribution is a null one, and one whose robustness is now documented: every one of the 26 bilirubin estimates was null (nine model specifications: p 0.109 to 0.436), including baseline-adjusted, doubly robust and inverse-probability-of-observation-weighted models. This is a specification-robust null, not a fragile one, but it is a null of limited resolution: the cohort could only detect a 0.11 mg/dL/day group-by-day difference at 80% power. These data describe an association between recorded medication exposure and subsequent biomarker trajectories. They do not compare continuation with discontinuation after admission, and they cannot establish that recorded exposure represents uninterrupted pre-admission therapy in every patient (a pre-admission record was documented in 60.0% of the exposed arm); they therefore provide no basis for a decision to continue or withhold either agent in an individual septic patient.

The between-arm difference in linear ALP slope, a steeper ALP rise in the exposed arm, is reported as hypothesis-generating, and we do not interpret it as drug-induced cholestasis. On formal robustness testing across 28 specifications, 12 retained p < 0.05. The signal survived log transformation, adjustment for the analyte's own day-1 value, doubly robust adjustment for source database and residual imbalances, addition of source database as a covariate, adjustment for concomitant glucose-lowering therapy, and exclusion of patients with coded chronic liver disease, which strengthened it. It did not survive allowing per-patient slopes for day, relaxing linearity in day, restricting exposure to records documented before admission, or omitting day-1 eGFR from the propensity model; and no single database reached significance in isolation (MIMIC-IV p = 0.054; eICU-CRD p = 0.328). Formal between-database heterogeneity was negligible (I^2^ 5.1%, Cochran Q p = 0.305), but statistical significance of the pooled estimate depended on the inclusion of MIMIC-IV and on the choice of fixed-versus random-effects pooling (fixed-effect p = 0.036; random-effects 95% CI −0.001 to +0.051). The defensible claim is therefore narrower than the primary estimate implies: a between-arm difference in linear ALP slope, robust to scale, baseline level, measured confounders and recognised liver disease, but dependent on propensity-score conditioning on renal function, the random-intercept-only covariance structure and on the assumption of linearity in day. Serum ALP is not liver-specific: the bone isoenzyme contributes substantially, and neither ALP isoenzyme fractionation nor gamma-glutamyl transferase is available in any of the three databases, so a hepatic origin cannot be established from these data. Diabetes duration, cumulative glucocorticoid exposure and bone disease were not measured in this cohort, and we therefore make no claim that they explain the finding; we note only that they are among the mechanisms by which ALP may rise without hepatic injury, and that nothing in these data excludes them. It is equally plausible, and equally unexcludable here, that DPP-4 inhibitors are preferentially prescribed to patients with pre-existing hepatic or biliary disease, which would generate this pattern with no pharmacodynamic effect; persistence of the signal after exclusion of coded chronic liver disease argues against this for recognised, though not for subclinical, liver disease. Three further considerations bear on interpretation. First, the day-7 denominator was 11 exposed and 37 comparator patients (89.5% and 87.5% missing respectively), and a seven-day slope anchored on that denominator is compatible with informative observation: the contrast was absent among survivors to discharge and when follow-up was truncated at day 4, although inverse-probability-of-observation weighting did not reduce the point estimate. Second, the ALP effect was located specifically in the linear day term of the random-intercept-only specification, a pattern consistent both with a slow cumulative pharmacodynamic effect and with progressive informative dropout selecting for higher-ALP survivors; the data do not distinguish these interpretations. Third, the contrast depends on including day-1 eGFR in the propensity model; because eGFR is partly a post-baseline covariate (Section 2.4), whether that conditioning removes confounding by indication or introduces bias cannot be resolved from these data. The absolute magnitude, roughly 49 U/L over a week, is at the margin of clinical relevance.

The concurrent MELD-Na divergence does not corroborate the ALP finding and is better read as a severity signal than a hepatic one. MELD-Na is a function of bilirubin, INR, creatinine and sodium; with the bilirubin term null, the divergence necessarily arises from the renal, coagulation or electrolyte components, and creatinine in this setting is dominated by septic acute kidney injury, which lies on the causal path between illness severity and outcome and was partially conditioned upon by the eGFR matching variable [19]. We therefore report MELD-Na descriptively and draw no hepatic inference from it.

### 4.1 Limitations

Interpretation of any between-arm difference is constrained by confounding by indication that matching on five covariates cannot resolve. Metformin is first-line therapy, whereas a DPP-4 inhibitor is characteristically what follows metformin failure, intolerance or contraindication. Diabetes duration, glycated haemoglobin and prior insulin use are absent from the propensity model and are precisely the variables separating the arms; residual confounding is therefore likely substantial and directionally predictable, with the DPP-4 inhibitor arm further into the disease course. Pre-sepsis glycaemic control, statin and renin–angiotensin–system inhibitor use, other potentially hepatotoxic co-medication and the anatomical source of sepsis are likewise unrecorded. The higher sulfonylurea co-exposure (43.8% versus 27.6%) and the residual imbalances in albumin, fibrinogen, aPTT, chronic liver disease and heart failure are consistent with an exposed arm further along the treatment pathway. These variables were carried into the doubly robust outcome models where measured, and the E-value analyses quantify how strong an unmeasured confounder would need to be to account for the positive findings; neither device substitutes for measurement. The DPP-4 inhibitor arm was overwhelmingly sitagliptin (82.9%) and linagliptin (13.3%), with only 4 saxagliptin users, so agent-level heterogeneity cannot be a substantial explanation of the between-arm signals here. In particular, the saxagliptin heart-failure signal cannot be driving the mortality difference at n = 4. The prevalent-user design also carries immortal-time and healthy-user bias [20].

Day-1 eGFR, drawn during established sepsis, is part chronic renal function and part acute kidney injury, so matching on it conditions on a partially post-baseline covariate that may lie on a causal path between severity and outcome; the 24-h SOFA score is open to the same objection. The primary specification should be read alongside the sensitivity analyses that omit day-1 eGFR, and that omit both eGFR and SOFA, from the propensity model (Supplementary Table S4): the bilirubin null holds under all three specifications, whereas the ALP contrast does not survive omission of eGFR. Sepsis and type 2 diabetes were identified from diagnosis codes and, in eICU-CRD, structured diagnosis entries rather than from clinical criteria; code-based sepsis identification favours specificity over sensitivity, carries no onset time in MIMIC-IV and NWICU, and the eICU-CRD code route for diabetes does not distinguish diabetes type. Exposure timing is the central limitation of this analysis. A pre-admission record was documented for 60.0% of exposed patients; the remaining 42 had a first record written during the index admission and cannot be classified as unequivocal pre-admission users. The primary analysis is therefore of recorded rather than verified pre-admission exposure, and admits both immortal-time bias and reverse causation. In particular, inpatient prescribing may respond to severity, renal function, oral intake, and evolving clinical status. eICU-CRD contributes 55.7% of the matched cohort, and its exposure ascertainment rests on an admission medication-reconciliation list that is self-reported and unverifiable against a dispensing record. Observation across days 1–7 is likewise informative rather than random, with day-7 denominators of 11 exposed and 37 comparator patients for ALP, and the analyses addressing this (inverse-probability-of-observation weighting, restriction to patients with at least three measurements or to survivors, and truncation at day 4) rest on assumptions that cannot be verified from these data. The mortality comparison was underpowered, since a 7.5 percentage-point difference at p = 0.083 is unresolved rather than null, and these data should not support a safety claim in an individual patient. The crude excess attenuated with documented pre-admission exposure and was concentrated where exposure was first recorded during the admission, so the mortality findings are exploratory and non-causal. The exposed arm was also too small for a sex-stratified analysis.

### 4.2 Directions for future work

The mechanistic hypothesis that motivates comparative work on incretin-axis therapies in sepsis belongs primarily to the GLP-1 receptor agonists and to the dual GIP/GLP-1 receptor agonist tirzepatide, which suppress NF-κB and NLRP3-inflammasome signalling, improve gut-barrier function, and for which lower sepsis- and infection-related mortality has been reported against DPP-4 inhibitor comparators [8], [9], [10], [21]. A DPP-4 inhibitor raises endogenous incretin concentrations only modestly and is not the leading candidate for a hepatoprotective effect during acute critical illness. Testing that hypothesis will require dispensing-registry linkage to hospital records, of the kind available in the Nordic health registries or the UK Clinical Practice Research Datalink, framed as a target-trial emulation with a new-user, active-comparator design, tirzepatide treated as a distinct exposure and mortality as a coprimary endpoint. Open critical-care databases, which hold no dispensing record and lack diabetes duration, glycated haemoglobin, gamma-glutamyl transferase and ALP isoenzyme fractionation, cannot support that design. The more immediate priority for the present question is a larger cohort with verifiable pre-admission exposure, which would resolve the mortality comparison this study leaves open and clarify whether the linear ALP slope difference reflects pharmacodynamics or the informative-observation pattern reported here.

## 5 Conclusion

In septic adults with type 2 diabetes, there was no statistically detectable difference between recorded DPP-4 inhibitor exposure and metformin-based therapy in hepatic biomarker trajectories, peak transaminases or platelet nadirs, although differences below the cohort's detectable-effect threshold cannot be excluded. The primary bilirubin null was robust across all nine specifications tested. A between-arm difference in linear ALP slope was present on the primary specification (+6.97 U/L/day; q = 0.005) but fragile on formal robustness testing (12 of 28 specifications retained p < 0.05); it survived scale, baseline-level and measured-confounder adjustment and exclusion of chronic liver disease but did not survive relaxation of the random-intercept-only covariance structure or of the linear day term, and is reported as hypothesis-generating. In-hospital mortality was numerically higher in the exposed arm (RR 1.56, 95% CI 0.97 to 2.49) but attenuated with documented pre-admission exposure (RR 1.23, 0.67 to 2.29); the mortality findings are exploratory and non-causal. These findings describe associations with recorded rather than verified pre-admission exposure, documented before admission in 60.0% of the exposed arm, and do not address whether these agents should be continued or withheld after admission. No change to the peri-critical-illness handling of these agents is warranted on their basis.

## CRediT authorship contribution statement

**Aristomenis G. Alevizopoulos:** Writing – review & editing, Writing – original draft, Visualization, Validation, Resources, Methodology, Investigation, Formal analysis, Data curation, Conceptualization. **Georgios G. Anastasiou:** Writing – original draft, Visualization, Software, Investigation, Data curation. **Paraskevas Nomikos:** Writing – review & editing, Software, Data curation. **Iakovos Kritikos:** Writing – review & editing, Validation, Supervision, Formal analysis, Data curation.

## Ethics declaration

The authors have NOT obtained informed consent from participants or their legal representatives. Not applicable. This is a retrospective secondary analysis of fully de-identified data from publicly available databases (MIMIC-IV, eICU-CRD, NWICU); no patient was contacted and no identifiable information was used. The requirement for informed consent was waived by the institutional review boards that approved the source databases (Beth Israel Deaconess Medical Center and Massachusetts Institute of Technology for MIMIC-IV; Northwestern University for NWICU), and eICU-CRD is exempt under HIPAA safe-harbor certification (no. 1031219-2).

This study was performed in compliance with relevant laws, regulatory frameworks and guidelines where the research took place. Ethics committee approval was not required under relevant laws and institutional guidelines. Secondary analysis of fully de-identified, publicly available data (MIMIC-IV, eICU-CRD, NWICU) accessed through PhysioNet under a credentialed data use agreement. Creation and research use of MIMIC-IV were approved by the institutional review boards of Beth Israel Deaconess Medical Center and the Massachusetts Institute of Technology, and NWICU by the Northwestern University institutional review board, each with a waiver of informed consent; eICU-CRD is exempt from institutional review board approval (HIPAA safe-harbor certification no. 1031219-2, Privacert). No additional approval was required for the present analysis.

## Data availability

The datasets analysed are available in the PhysioNet repository (https://physionet.org) subject to completion of the required training and credentialed data-use agreement. Analysis code is available from the corresponding author on reasonable request.

## Declaration of generative AI and AI-assisted technologies in the manuscript preparation process

Statement: During the preparation of this work the author(s) used Claude in order to improve editing and enhance the readability of the manuscript. After using this tool/service, the author(s) reviewed and edited the content as needed and take(s) full responsibility for the content of the published article.

## Funding

This research received no specific grant from any funding agency in the public, commercial or not-for-profit sectors.

## Competing interests

The authors declare that there is no conflict of interest.
