# Supplementary material for: Hepatic biomarker trajectories and mortality in septic adults with type 2 diabetes and recorded dipeptidyl peptidase-4 inhibitor exposure versus metformin-based therapy: A propensity-score-matched cohort study
Source: Metabol Open. 2026 Oct 10;32:100508. doi: 10.1016/j.metop.2026.100508 (PMC13678097; doi:10.1016/j.metop.2026.100508)
Supplement: Supplementary file 1 [file mmc1.docx]

**Supplementary Material**

*Hepatic biomarker trajectories and mortality in septic adults with type 2 diabetes with recorded dipeptidyl peptidase-4 inhibitor exposure versus metformin-based therapy: A Propensity-Score-Matched Cohort Study*

Manuscript METOP-S-26-00285 — Revised submission

All estimates are reported from the reviewer-response analysis runs (25 September and October 2026) on the matched cohort of 105 DPP-4 inhibitor and 297 metformin patients drawn from MIMIC-IV v3.1, eICU-CRD v2.0 and NWICU v0.1.0. Machine-readable versions of every table are provided in the accompanying workbook (Supplementary_Material_revision.xlsx). Log-scale estimates are group × day interactions on log(x+1)-transformed values, in log units per day.

## **Contents**

**Section 1. Cohort definitions**

**Table S0a** Operational definitions of sepsis, type 2 diabetes and the analysis unit in each database.

**Table S0b**	Codes that qualified the matched patients, MIMIC-IV and NWICU.

**Table S0c** Verification that the code-based definitions behaved identically in MIMIC-IV and NWICU.

**Section 2. Covariate balance**

**Table S1**	Standardised mean differences and arm means for every measured covariate, before (1,623 eligible) and after…

**Figure S1** Love plot of absolute standardised mean differences before (open circles) and after (filled circles) matchi…

**Section 3. Exposure ascertainment and timing**

**Table S2a**	Exposure ascertainment by source database.

**Table S2b**	Timing of the first DPP-4 inhibitor record, by source database.

**Table S2c** Distribution of the first-record timestamp relative to admission, by source database.

**Table S2d**	DPP-4 inhibitor agents in the exposed arm (n=105).

**Section 4. Matched-set structure and database heterogeneity**

**Table S3**	Database-specific cohort summary before and after matching.

**Table S3a**	Matched-set structure.

**Table S3b** Database-specific group-by-day interaction estimates, log(x+1) scale.

**Table S3c** Between-database heterogeneity and random-effects pooling (DerSimonian–Laird), log(x+1) scale.

**Table S3d**	Leave-one-database-out estimates, log(x+1) scale.

**Figure S3d** Leave-one-database-out estimates for total bilirubin (top) and ALP (bottom), log(x+1) scale. Point estimate…

**Table S3e** Admission year by source database and arm (de-identified, date-shifted years).

**Section 5. Propensity-score specification, estimand and exposure-window sensitivity**

**Table S4a**	Matched cohort under three propensity-score specifications.

**Table S4**	Outcome estimates under three propensity-score specifications, log(x+1) scale.

**Figure S4** Outcome estimates under three propensity-score specifications, log(x+1) scale. Point estimates with 95% con…

**Table S4b**	Estimand and exposure-window sensitivity analyses, log(x+1) scale.

**Figure S4b** Estimand and exposure-window sensitivity analyses, log(x+1) scale. Point estimates with 95% confidence inte…

**Table S4c** Post-matching standardised mean differences under each propensity-score specification.

**Section 6. Model specification, multiplicity and informative observation**

**Table S5**	Group × day interaction under ten model specifications.

**Figure S5** Group × day interaction under the model specifications with a single point estimate: raw scale (units per d…

**Table S5b** All trajectory outcomes with Benjamini–Hochberg correction across the full family, log(x+1) scale.

**Table S6**	Patients contributing a value on each study day, by analyte and arm.

**Figure S6** Proportion of each arm contributing a measurement on days 1–7, by analyte. Attrition curves are near-parall…

**Figure S9** Daily trajectories of albumin, INR, aPTT, platelets, lactate, MELD-Na, hepatic SOFA subscore and FIB-4 over…

**Table S6b** Observation model: predictors of contributing a measurement on a given day.

**Table S6c**	Informative-observation sensitivity analyses, log(x+1) scale.

**Figure S6c** Informative-observation sensitivity analyses, log(x+1) scale. Point estimates with 95% confidence intervals…

**Section 7. Mortality**

**Table S7a** Mortality by endpoint, with risk ratios, confidence intervals and E-values.

**Figure S7a** Risk ratios for mortality, DPP-4 inhibitor versus metformin, with 95% confidence intervals (log scale): in-…

**Table S7b**	In-hospital mortality accounting for the matched design.

**Table S7c**	Mortality ascertainment by source database.

**Table S7e** In-hospital mortality restricted to documented pre-admission exposure.

**Section 8. Full trajectory model output, specification summaries, peak/nadir outcomes, minimum detectable effects and E-values**

**Table S8**	Primary raw-scale mixed-model output for every trajectory analyte (the full version of Table 2).

**Table S8a**	Every ALP estimate produced for the revision.

**Table S8b** Every total-bilirubin (primary outcome) estimate produced for the revision.

**Figure S8b** Every total-bilirubin (primary outcome) estimate produced for the revision: raw-scale specifications (top) …

**Table S9a** Minimum detectable group × day effect at 80% power and α = 0.05, raw scale.

**Table S9b** Minimum detectable in-hospital mortality effect at 80% power and α = 0.05.

**Table S9c**	E-values for the reported positive findings.

**Table S10**	Peak and nadir values over study days 1–7.

# **Section 1. Cohort definitions**

*Addresses Reviewer 2 round-2 Comment 1 (operational criteria for sepsis and type 2 diabetes in each database).*

**Table S0a.** Operational definitions of sepsis, type 2 diabetes and the analysis unit in each database.

| **Database** | **Element** | **Source table and field** | **Definition** | **Timing** |
| --- | --- | --- | --- | --- |
| MIMIC-IV and NWICU | Sepsis | hosp.diagnoses_icd (icd_version, icd_code) | ICD-9-CM 99591, 99592, 78552; ICD-10-CM A41 (any subcode), R6520, R6521, A021, A227, A267, A327, A427 | Discharge diagnosis of the index hospital admission; no onset time |
| MIMIC-IV and NWICU | Type 2 diabetes | hosp.diagnoses_icd (icd_version, icd_code) | ICD-9-CM 250x0 or 250x2 (fifth digit 0 or 2); ICD-10-CM E11 (any subcode) | Same hospital admission as the sepsis code |
| MIMIC-IV and NWICU | Analysis unit | hosp.admissions | First admission per patient carrying both a sepsis and a type 2 diabetes code; age ≥18 years | — |
| eICU-CRD | Sepsis | diagnosis.diagnosisstring; patient.apacheadmissiondx | Diagnosis entry containing ‘sepsis’, ‘septic shock’, ‘septicemia’ or ‘severe sepsis’; or an APACHE admission diagnosis containing these terms | Diagnosis entry with diagnosisoffset ≤1,440 min (24 h) from unit admission; APACHE diagnosis at admission |
| eICU-CRD | Type 2 diabetes | diagnosis.diagnosisstring; diagnosis.icd9code | Diagnosis entry containing ‘diabetes mellitus’ or ‘type 2 (II) diabetes’ and not ‘type 1 (I)’ or ‘insulin-dependent’; or an icd9code field containing 250.x or E11 | Any time during the unit stay |
| eICU-CRD | Analysis unit | patient | First unit stay (unitvisitnumber = 1) with unit length of stay ≥24 h; one stay per hospital admission; age ≥18 years | — |

*Sepsis and type 2 diabetes were identified from diagnosis codes and, in eICU-CRD, structured diagnosis entries; clinical Sepsis-3 criteria were not applied. MIMIC-IV and NWICU share the MIMIC-IV table structure and were processed by the same query, so their definitions are identical. In eICU-CRD, because ‘non-insulin-dependent’ contains the excluded term ‘insulin-dependent’, entries worded that way do not qualify through the text route; such patients qualify only through the ICD route. The eICU-CRD ICD route (250.x) does not distinguish diabetes type.*

**Table S0b.** Codes that qualified the matched patients, MIMIC-IV and NWICU.

| **Database** | **Criterion** | **Qualifying codes (matched patients)** |
| --- | --- | --- |
| MIMIC-IV | Sepsis | ICD-10 A419 (48); ICD-9 99592 (37); ICD-10 R6521 (34); ICD-9 78552 (16); ICD-10 R6520 (15); ICD-9 99591 (14); ICD-10 A4189 (12); ICD-10 A4151 (11); ICD-10 A4101 (4); ICD-10 A4181 (4); ICD-10 A4102 (3); ICD-10 A4150 (2); ICD-10 A4159 (2); ICD-10 A411 (2); ICD-10 A414 (1); ICD-10 A4152 (1) |
| MIMIC-IV | Type 2 diabetes | ICD-10 E1165 (32); ICD-9 25000 (32); ICD-10 E1122 (31); ICD-10 E119 (28); ICD-10 E1151 (11); ICD-10 E1142 (10); ICD-10 E1140 (9); ICD-10 E11319 (6); ICD-9 25002 (5); ICD-10 E11649 (5); ICD-9 25062 (5); ICD-10 E11621 (4); ICD-10 E1110 (4); ICD-10 E1169 (4); ICD-10 E1152 (3); ICD-9 25060 (2); ICD-9 25012 (2); ICD-9 25052 (2); ICD-10 E11339 (1); ICD-9 25040 (1); ICD-10 E118 (1); ICD-9 25082 (1); ICD-9 25080 (1); ICD-10 E1139 (1); ICD-10 E11628 (1); ICD-10 E11610 (1); ICD-9 25042 (1); ICD-9 25090 (1); ICD-9 25092 (1) |
| NWICU | Sepsis | ICD-10 A419 (34); ICD-10 R6520 (11); ICD-10 R6521 (7); ICD-10 A4151 (3); ICD-10 A4189 (2); ICD-10 A4150 (2) |
| NWICU | Type 2 diabetes | ICD-10 E119 (19); ICD-10 E1165 (12); ICD-10 E1110 (3); ICD-10 E11628 (2); ICD-10 E1129 (1); ICD-10 E1169 (1); ICD-10 E11621 (1); ICD-10 E1159 (1); ICD-10 E11649 (1); ICD-10 E1122 (1) |

*Codes are shown as stored, without the decimal point (e.g. A419 = A41.9). A patient can carry more than one qualifying code, so counts within a row can exceed the number of matched patients. NWICU records ICD-10 only. eICU-CRD is not shown because most of its patients qualified through diagnosis entries rather than codes.*

**Table S0c.** Verification that the code-based definitions behaved identically in MIMIC-IV and NWICU.

| **Database** | **Sepsis + T2DM admissions (as run)** | **Patients (as run)** | **Admissions (format-tolerant)** | **Patients (format-tolerant)** | **Patients missed by code formatting** |
| --- | --- | --- | --- | --- | --- |
| MIMIC-IV | 6,916 | 5,593 | 6,916 | 5,593 | 0 |
| NWICU | 441 | 424 | 441 | 424 | 0 |

*The as-run query was repeated with code formatting normalised (decimal points and spaces removed, ICD version read as text). Identical counts show that no eligible admission was lost to differences in how either database stores diagnosis codes. Counts are before exposure classification and matching.*

# **Section 2. Covariate balance**

*Addresses Reviewer 2 Major 4 and Reviewer 3 Point 3.*

**Table S1.** Standardised mean differences and arm means for every measured covariate, before (1,623 eligible) and after (402 matched) propensity-score matching.

| **Covariate** | **DPP-4i mean, pre** | **Metformin mean, pre** | **SMD pre** | **DPP-4i mean, post** | **Metformin mean, post** | **SMD post** | **\|SMD\|<0.10 post** |
| --- | --- | --- | --- | --- | --- | --- | --- |
| Age (years) * | 71.405 | 67.390 | 0.312 | 71.524 | 71.468 | 0.004 | Yes |
| Body mass index (kg/m²) * | 31.345 | 31.373 | -0.003 | 31.388 | 31.199 | 0.021 | Yes |
| Male sex (proportion) * | 0.569 | 0.551 | 0.037 | 0.543 | 0.549 | -0.012 | Yes |
| 24-hour SOFA score * | 2.707 | 3.107 | -0.124 | 2.867 | 2.953 | -0.028 | Yes |
| Day-1 eGFR (mL/min/1.73 m²) * | 47.629 | 62.596 | -0.495 | 48.656 | 49.906 | -0.043 | Yes |
| Creatinine (mg/dL) | 1.898 | 1.520 | 0.302 | 1.849 | 1.901 | -0.037 | Yes |
| BUN (mg/dL) | 36.943 | 30.361 | 0.273 | 36.507 | 37.066 | -0.022 | Yes |
| Sodium (mmol/L) | 137.691 | 136.851 | 0.156 | 137.747 | 136.404 | 0.246 | No |
| Potassium (mmol/L) | 4.305 | 4.194 | 0.157 | 4.281 | 4.241 | 0.060 | Yes |
| Heart failure | 0.302 | 0.282 | 0.043 | 0.305 | 0.239 | 0.148 | No |
| Coronary artery disease | 0.284 | 0.312 | -0.060 | 0.286 | 0.242 | 0.098 | Yes |
| COPD | 0.138 | 0.139 | -0.002 | 0.124 | 0.135 | -0.032 | Yes |
| Chronic liver disease | 0.069 | 0.119 | -0.173 | 0.076 | 0.040 | 0.153 | No |
| Day-1 albumin (g/dL) | 3.002 | 2.982 | 0.030 | 2.975 | 2.756 | 0.343 | No |
| Day-1 total bilirubin (mg/dL) | 0.868 | 1.211 | -0.187 | 0.869 | 1.160 | -0.213 | No |
| Day-1 ALP (U/L) | 126.180 | 128.195 | -0.018 | 127.200 | 117.382 | 0.103 | No |
| Day-1 ALT (U/L) | 73.910 | 84.129 | -0.041 | 74.608 | 70.201 | 0.025 | Yes |
| Day-1 AST (U/L) | 153.320 | 121.512 | 0.055 | 155.425 | 93.219 | 0.170 | No |
| Day-1 INR | 1.531 | 1.696 | -0.182 | 1.543 | 1.658 | -0.143 | No |
| Day-1 platelets (×10⁹/L) | 215.849 | 228.273 | -0.104 | 213.194 | 215.242 | -0.017 | Yes |
| Day-1 fibrinogen (mg/dL) | 435.250 | 508.903 | -0.345 | 435.250 | 489.348 | -0.277 | No |
| Day-1 aPTT (s) | 36.218 | 36.801 | -0.036 | 34.368 | 37.894 | -0.235 | No |
| MELD-Na | 16.418 | 16.619 | -0.027 | 16.422 | 18.263 | -0.244 | No |
| FIB-4 index | 5.304 | 4.661 | 0.078 | 5.347 | 5.270 | 0.009 | Yes |
| Hepatic SOFA subscore | 0.262 | 0.378 | -0.165 | 0.267 | 0.374 | -0.150 | No |
| Sulfonylurea | 0.448 | 0.324 | 0.258 | 0.438 | 0.276 | 0.343 | No |
| Thiazolidinedione | 0.069 | 0.032 | 0.170 | 0.076 | 0.024 | 0.244 | No |
| SGLT2 inhibitor | 0.017 | 0.015 | 0.021 | 0.019 | 0.003 | 0.149 | No |
| Meglitinide | 0.026 | 0.015 | 0.080 | 0.029 | 0.003 | 0.202 | No |
| Basal insulin | 0.250 | 0.130 | 0.309 | 0.219 | 0.232 | -0.032 | Yes |
| Prandial insulin | 0.310 | 0.093 | 0.563 | 0.257 | 0.199 | 0.140 | No |

** Variable in the propensity model. Binary variables are shown as proportions. Balance judged on |SMD|<0.10; every covariate at or above that threshold after matching is carried into the doubly robust outcome models (Methods 2.4) and into the concomitant-therapy adjustment in Table S4b (row E). Missing-value counts per covariate are given in the accompanying workbook (sheet S1).*

**Figure S1.** Love plot of absolute standardised mean differences before (open circles) and after (filled circles) matching. Dashed line at |SMD| = 0.10. The five propensity-model covariates are balanced after matching; covariates outside the propensity model that remain above the threshold are adjusted for in the outcome models.

# **Section 3. Exposure ascertainment and timing**

*Addresses Reviewer 2 Major 1, Major 2 and Minor 4, and Reviewer 3 Point 3.*

**Table S2a.** Exposure ascertainment by source database.

| **Database** | **Table** | **Field matched** | **Coding system** | **Timing field** | **Reference point** | **Captures pre-admission therapy?** |
| --- | --- | --- | --- | --- | --- | --- |
| MIMIC-IV v3.1 | hosp/prescriptions | drug (free text) | none — case-insensitive regex on generic + brand names | starttime | hospital admittime and ICU intime | Only where an order was written before admittime; MIMIC-IV holds no outpatient dispensing record. |
| NWICU v0.1.0 | hosp/prescriptions (MIMIC-style layout) | drug (free text) | none — same regex as MIMIC-IV | starttime | hospital admittime and ICU intime | Same as MIMIC-IV. |
| eICU-CRD v2.0 | admissiondrug (EICU_EXPOSURE_SOURCE=admissiondrug) | drugname (free text) | none — same regex; drughiclseqno not used | drugoffset (minutes from unit admission), drugenteredoffset | unit admission (offset 0) | admissiondrug IS the admission medication-reconciliation list, i.e. therapy reported as taken before arrival. It is self-reported and cannot be verified against a dispensing record. |

*The same case-insensitive regular expression (generic and brand names of sitagliptin, saxagliptin, linagliptin, alogliptin and vildagliptin, including fixed-dose combinations) was applied in all three databases.*

**Table S2b.** Timing of the first DPP-4 inhibitor record, by source database.

| **Database** | **Exposed, n** | **Pre-admission record (primary definition)** | **Timestamp at or before time zero (strict)** | **First record at or before first bilirubin** | **Hours from admission, median (IQR)** |
| --- | --- | --- | --- | --- | --- |
| eICU-CRD | 59 | 59 (100.0%) | 11 (18.6%) | 25 (42.4%) | 0.7 (0.3 to 1.8) |
| MIMIC-IV | 35 | 2 (5.7%) | 2 (5.7%) | 12 (34.3%) | 40.5 (19.1 to 124.2) |
| NWICU | 11 | 2 (18.2%) | 2 (18.2%) | 11 (100.0%) | 14.8 (7.1 to 16.5) |
| All databases | 105 | 63 (60.0%) | 15 (14.3%) | 48 (45.7%) | — |

*Primary definition (column 3): in eICU-CRD, any entry in the admission medication-reconciliation list (admissiondrug), which by construction records therapy reported as taken before arrival and is entered in the first hours of the unit stay; in MIMIC-IV and NWICU, a prescription with starttime before hospital admittime. Column 4 applies the strict timestamp criterion to all sources, including eICU-CRD reconciliation entries. Column 5 counts records dated at or before the first total-bilirubin measurement of the index stay. For eICU-CRD, hours are measured from unit admission (drugoffset); for MIMIC-IV and NWICU from hospital admittime.*

**Table S2c.** Distribution of the first-record timestamp relative to admission, by source database.

| **Database** | **n with timestamp** | **Median (h)** | **Q1 (h)** | **Q3 (h)** | **Min (h)** | **Max (h)** | **At/before 0 h** | **Within 24 h** | **After 24 h** | **Reference point** |
| --- | --- | --- | --- | --- | --- | --- | --- | --- | --- | --- |
| eICU-CRD | 59 | 0.7 | 0.3 | 1.8 | -2.4 | 20.0 | 11 | 48 | 0 | admissiondrug drugoffset (min from unit admission) |
| MIMIC-IV | 35 | 40.5 | 19.1 | 124.2 | -8.0 | 685.5 | 2 | 11 | 22 | prescriptions.starttime - admissions.admittime (h) |
| NWICU | 11 | 14.8 | 7.1 | 16.5 | -33.2 | 61.1 | 2 | 8 | 1 | prescriptions.starttime - admissions.admittime (h) |

**Table S2d.** DPP-4 inhibitor agents in the exposed arm (n=105).

| **Agent** | **Exposed patients, n** | **% of DPP-4 inhibitor arm** |
| --- | --- | --- |
| Sitagliptin | 87 | 82.9 |
| Saxagliptin | 4 | 3.8 |
| Linagliptin | 14 | 13.3 |
| Alogliptin | 0 | 0.0 |
| Vildagliptin | 0 | 0.0 |

*The arm is 96.2% sitagliptin or linagliptin. With four saxagliptin users, the saxagliptin heart-failure signal cannot account for the between-arm mortality difference.*

# **Section 4. Matched-set structure and database heterogeneity**

*Addresses Reviewer 2 Major 5 and Major 12.*

**Table S3.** Database-specific cohort summary before and after matching.

| **Database** | **Eligible** | **DPP-4i pre** | **Metformin pre** | **DPP-4i matched** | **Metformin matched** | **% of matched cohort** | **Mean age** | **Mean 24-h SOFA** | **In-hospital death, DPP-4i %** | **In-hospital death, metformin %** | **Post-discharge vital status** |
| --- | --- | --- | --- | --- | --- | --- | --- | --- | --- | --- | --- |
| eICU-CRD | 397 | 62 | 335 | 59 | 165 | 55.7 | 70.4 | 2.74 | 18.6 | 15.8 | no |
| MIMIC-IV | 1171 | 35 | 1136 | 35 | 105 | 34.8 | 74.9 | 4.03 | 31.4 | 13.3 | yes |
| NWICU | 55 | 19 | 36 | 11 | 27 | 9.5 | 65.3 | 0.00 | 0.0 | 0.0 | no |
| All | 1,623 | 116 | 1,507 | 105 | 297 | 100 | 71.5 | — | 21.0 | 13.5 | MIMIC-IV only |

*SOFA in NWICU could not be reconstructed from the available fields and is recorded as 0; matching on SOFA within NWICU is therefore uninformative, which is one reason matching was performed within database.*

**Table S3a.** Matched-set structure.

| **Comparators per exposed patient** | **Matched sets, n** | **% of sets** |
| --- | --- | --- |
| 1 | 6 | 5.7 |
| 2 | 6 | 5.7 |
| 3 | 93 | 88.6 |
| Total | 105 | 100.0 |

*105 matched sets; 297 comparators; mean ratio 2.83:1. Eleven of 116 exposed patients had no comparator within the caliper and were not matched.*

**Table S3b.** Database-specific group-by-day interaction estimates, log(x+1) scale.

| **Analyte** | **Database** | **Estimate** | **95% CI** | **p** | **Patients** | **Observations** |
| --- | --- | --- | --- | --- | --- | --- |
| Total Bilirubin | eICU-CRD | 0.0093 | -0.0088 to 0.0274 | 0.315 | 188 | 463 |
| Total Bilirubin | MIMIC-IV | 0.0193 | -0.0170 to 0.0555 | 0.298 | 107 | 336 |
| Total Bilirubin | NWICU | — | — | — | — | Did not converge (insufficient repeated measurements) |
| ALP | eICU-CRD | 0.0153 | -0.0154 to 0.0460 | 0.328 | 190 | 467 |
| ALP | MIMIC-IV | 0.0435 | -0.0008 to 0.0878 | 0.054 | 105 | 330 |
| ALP | NWICU | — | — | — | — | Did not converge (insufficient repeated measurements) |

**Table S3c.** Between-database heterogeneity and random-effects pooling (DerSimonian–Laird), log(x+1) scale.

| **Analyte** | **Databases pooled** | **RE pooled estimate** | **95% CI** | **I² (%)** | **Cochran Q** | **p (Q)** |
| --- | --- | --- | --- | --- | --- | --- |
| Total Bilirubin | 2 | 0.0113 | -0.0049 to 0.0275 | 0.0 | 0.233 | 0.629 |
| ALP | 2 | 0.0247 | -0.0014 to 0.0508 | 0.1 | 1.054 | 0.304 |

*Heterogeneity is negligible for both analytes. NWICU did not contribute because its models did not converge.*

**Table S3d.** Leave-one-database-out estimates, log(x+1) scale.

| **Analyte** | **Analysis** | **Group × day (95% CI); p** | **Patients** | **Observations** | **p < 0.05** |
| --- | --- | --- | --- | --- | --- |
| Total Bilirubin | all databases | +0.014 (-0.003 to +0.032); p=0.1123 | 295 | 799 | No |
| Total Bilirubin | excluding eICU-CRD | +0.019 (-0.017 to +0.056); p=0.2979 | 107 | 336 | No |
| Total Bilirubin | excluding MIMIC-IV | +0.009 (-0.009 to +0.027); p=0.3152 | 188 | 463 | No |
| Total Bilirubin | excluding NWICU | +0.014 (-0.003 to +0.032); p=0.1123 | 295 | 799 | No |
| ALP | all databases | +0.027 (+0.002 to +0.052); p=0.0358 | 295 | 797 | Yes |
| ALP | excluding eICU-CRD | +0.043 (-0.001 to +0.088); p=0.0541 | 105 | 330 | No |
| ALP | excluding MIMIC-IV | +0.015 (-0.015 to +0.046); p=0.3284 | 190 | 467 | No |
| ALP | excluding NWICU | +0.027 (+0.002 to +0.052); p=0.0358 | 295 | 797 | Yes |

*Excluding NWICU leaves estimates unchanged because NWICU did not contribute repeated measurements to either model.*

**Figure S3d.** Leave-one-database-out estimates for total bilirubin (top) and ALP (bottom), log(x+1) scale. Point estimates with 95% confidence intervals for the group × day interaction. Orange: p<0.05; teal: p≥0.05. Dashed line: no difference in daily slope between arms. Excluding NWICU reproduces the all-database estimate because NWICU contributed no repeated measurements.

**Table S3e.** Admission year by source database and arm (de-identified, date-shifted years).

| **Database** | **Arm** | **Minimum** | **Median** | **Maximum** | **n** |
| --- | --- | --- | --- | --- | --- |
| eICU-CRD | DPP-4i | Not recorded | Not recorded | Not recorded | 0 |
| eICU-CRD | Metformin | Not recorded | Not recorded | Not recorded | 0 |
| MIMIC-IV | DPP-4i | 2116 | 2157 | 2198 | 35 |
| MIMIC-IV | Metformin | 2111 | 2160 | 2204 | 105 |
| NWICU | DPP-4i | 2115 | 2152 | 2197 | 11 |
| NWICU | Metformin | 2101 | 2158 | 2198 | 27 |

*MIMIC-IV and NWICU shift each patient's dates into the future by a patient-specific offset, so the years shown are not calendar years; they are nonetheless comparable between arms within a database. eICU-CRD releases no admission year. Calendar time is therefore represented in the outcome models by the source-database term (which is itself tied to the collection period: eICU-CRD 2014–2015, MIMIC-IV 2008–2022, NWICU 2013–2023), and the arms are comparable in shifted year within MIMIC-IV and NWICU.*

# **Section 5. Propensity-score specification, estimand and exposure-window sensitivity**

*Addresses Reviewer 2 Majors 2, 3, 4 and 9.*

**Table S4a.** Matched cohort under three propensity-score specifications.

| **PS specification** | **PS variables** | **DPP4 matched** | **METFORMIN matched** | **total matched** | **matched sets** |
| --- | --- | --- | --- | --- | --- |
| published (age, bmi, male, SOFA, eGFR) | age, bmi, male, sofa_24h, egfr | 105 | 297 | 402 | 105 |
| eGFR omitted (age, bmi, male, SOFA) | age, bmi, male, sofa_24h | 105 | 300 | 405 | 105 |
| eGFR and SOFA omitted (age, bmi, male) | age, bmi, male | 104 | 299 | 403 | 104 |

**Table S4.** Outcome estimates under three propensity-score specifications, log(x+1) scale.

| **Analyte** | **Propensity-score specification** | **Group × day (95% CI); p** | **Patients** | **Observations** | **p < 0.05** |
| --- | --- | --- | --- | --- | --- |
| Total Bilirubin | published (age, bmi, male, SOFA, eGFR) | +0.014 (-0.003 to +0.032); p=0.1123 | 295 | 799 | No |
| ALP | published (age, bmi, male, SOFA, eGFR) | +0.027 (+0.002 to +0.052); p=0.0358 | 295 | 797 | Yes |
| Total Bilirubin | eGFR omitted (age, bmi, male, SOFA) | -0.004 (-0.023 to +0.015); p=0.6761 | 293 | 813 | No |
| ALP | eGFR omitted (age, bmi, male, SOFA) | +0.007 (-0.021 to +0.034); p=0.6303 | 294 | 804 | No |
| Total Bilirubin | eGFR and SOFA omitted (age, bmi, male) | +0.010 (-0.010 to +0.030); p=0.3262 | 288 | 778 | No |
| ALP | eGFR and SOFA omitted (age, bmi, male) | +0.007 (-0.019 to +0.032); p=0.6116 | 285 | 773 | No |

*Omitting day-1 eGFR (and 24-hour SOFA) tests whether conditioning on partially post-baseline covariates drives the estimates (Methods 2.4). The bilirubin null holds under every specification. Balance under each specification is given in Table S4c.*

**Figure S4.** Outcome estimates under three propensity-score specifications, log(x+1) scale. Point estimates with 95% confidence intervals for the group × day interaction. Orange: p<0.05; teal: p≥0.05. Dashed line: no difference in daily slope between arms.

**Table S4b.** Estimand and exposure-window sensitivity analyses, log(x+1) scale.

| **Analyte** | **Analysis** | **Group × day (95% CI); p** | **Patients** | **Observations** | **p < 0.05** |
| --- | --- | --- | --- | --- | --- |
| Total Bilirubin | A. all recorded exposure (published) | +0.014 (-0.003 to +0.032); p=0.1123 | 295 | 799 | No |
| ALP | A. all recorded exposure (published) | +0.027 (+0.002 to +0.052); p=0.0358 | 295 | 797 | Yes |
| Total Bilirubin | B. exposure at/before time zero (61 exposed, 270 comparators) | +0.015 (-0.005 to +0.036); p=0.1448 | 269 | 734 | No |
| ALP | B. exposure at/before time zero (61 exposed, 270 comparators) | +0.022 (-0.006 to +0.050); p=0.1232 | 270 | 733 | No |
| Total Bilirubin | C. DPP-4i+metformin (n=45) vs metformin alone | +0.022 (-0.007 to +0.050); p=0.1312 | 250 | 683 | No |
| ALP | C. DPP-4i+metformin (n=45) vs metformin alone | +0.034 (-0.004 to +0.071); p=0.0827 | 250 | 679 | No |
| Total Bilirubin | D. DPP-4i without metformin (n=60) vs metformin alone | +0.010 (-0.012 to +0.031); p=0.3686 | 260 | 725 | No |
| ALP | D. DPP-4i without metformin (n=60) vs metformin alone | +0.024 (-0.005 to +0.052); p=0.1084 | 261 | 724 | No |
| Total Bilirubin | E. adjusted for concomitant therapy (ad_sulfonylurea, ad_basal_insulin, ad_prandial_insulin) | +0.015 (-0.003 to +0.032); p=0.1043 | 295 | 799 | No |
| ALP | E. adjusted for concomitant therapy (ad_sulfonylurea, ad_basal_insulin, ad_prandial_insulin) | +0.027 (+0.002 to +0.052); p=0.0354 | 295 | 797 | Yes |
| Total Bilirubin | F. excluding coded chronic liver disease (n=20 removed) | +0.011 (-0.007 to +0.030); p=0.2332 | 277 | 741 | No |
| ALP | F. excluding coded chronic liver disease (n=20 removed) | +0.033 (+0.006 to +0.060); p=0.0164 | 277 | 739 | Yes |

*B restricts exposure to records at or before time zero. C and D decompose the estimand by metformin co-exposure (Reviewer 2 Major 3). E adds sulfonylurea, basal-insulin and prandial-insulin co-exposure. F removes the 20 patients with coded chronic liver disease (Reviewer 2 Major 9); the ALP estimate is larger and more precise without them, so pre-existing liver disease does not account for the signal. The bilirubin null holds in every row.*

**Figure S4b.** Estimand and exposure-window sensitivity analyses, log(x+1) scale. Point estimates with 95% confidence intervals for the group × day interaction. Orange: p<0.05; teal: p≥0.05. Dashed line: no difference in daily slope between arms. The bilirubin estimate is null in every analysis; the ALP estimate is largest after excluding chronic liver disease.

**Table S4c.** Post-matching standardised mean differences under each propensity-score specification.

| **Covariate** | **SMD: published (age, bmi, male, SOFA, eGFR)** | **SMD: eGFR omitted (age, bmi, male, SOFA)** | **SMD: eGFR and SOFA omitted (age, bmi, male)** |
| --- | --- | --- | --- |
| Age (years) | 0.004 | -0.026 | -0.021 |
| Body mass index (kg/m²) | 0.021 | 0.007 | 0.110 |
| Male sex (proportion) | -0.012 | 0.033 | 0.008 |
| 24-hour SOFA score | -0.028 | -0.015 | 0.122 |
| Day-1 eGFR (mL/min/1.73 m²) | -0.043 | -0.159 | -0.310 |
| Creatinine (mg/dL) | -0.037 | 0.070 | 0.215 |
| BUN (mg/dL) | -0.022 | 0.064 | 0.210 |
| Sodium (mmol/L) | 0.246 | 0.151 | 0.062 |
| Potassium (mmol/L) | 0.060 | 0.109 | 0.134 |
| Heart failure | 0.148 | 0.113 | 0.211 |
| Coronary artery disease | 0.098 | 0.102 | 0.248 |
| COPD | -0.032 | -0.086 | 0.024 |
| Chronic liver disease | 0.153 | -0.063 | 0.157 |
| Day-1 albumin (g/dL) | 0.343 | 0.315 | 0.264 |
| Day-1 total bilirubin (mg/dL) | -0.213 | -0.157 | -0.244 |
| Day-1 ALP (U/L) | 0.103 | 0.094 | 0.177 |
| Day-1 ALT (U/L) | 0.025 | -0.080 | 0.114 |
| Day-1 AST (U/L) | 0.170 | 0.028 | 0.246 |
| Day-1 INR | -0.143 | -0.257 | -0.138 |
| Day-1 platelets (×10⁹/L) | -0.017 | 0.026 | -0.040 |
| Day-1 fibrinogen (mg/dL) | -0.277 | -0.055 | -0.531 |
| Day-1 aPTT (s) | -0.235 | -0.112 | 0.026 |
| MELD-Na | -0.244 | -0.217 | 0.014 |
| FIB-4 index | 0.009 | -0.023 | 0.098 |
| Hepatic SOFA subscore | -0.150 | -0.103 | -0.131 |
| Sulfonylurea | 0.343 | 0.361 | 0.361 |
| Thiazolidinedione | 0.244 | 0.231 | 0.247 |
| SGLT2 inhibitor | 0.149 | 0.110 | 0.111 |
| Meglitinide | 0.202 | 0.110 | 0.077 |
| Basal insulin | -0.032 | -0.026 | 0.001 |
| Prandial insulin | 0.140 | 0.117 | 0.171 |

# **Section 6. Model specification, multiplicity and informative observation**

*Addresses Reviewer 2 Majors 6, 7, 8 and 12.*

**Table S5.** Group × day interaction under ten model specifications.

| **Analyte** | **Specification** | **Group × day (95% CI); p** | **Patients** | **Observations** | **p < 0.05** | **Note** |
| --- | --- | --- | --- | --- | --- | --- |
| Total Bilirubin | 1. published (raw, linear, RI) | +0.048 (-0.029 to +0.126); p=0.2208 | 295 | 799 | No |  |
| Total Bilirubin | 2. log(x+1), linear, RI | +0.014 (-0.003 to +0.032); p=0.1123 | 295 | 799 | No |  |
| Total Bilirubin | 3. raw, linear, random slope | +0.050 (-0.051 to +0.151); p=0.3310 | 295 | 799 | No |  |
| Total Bilirubin | 4. log, linear, random slope | +0.019 (-0.007 to +0.044); p=0.1556 | 295 | 799 | No |  |
| Total Bilirubin | 5. log, day as factor (joint test) | joint p=0.4036 | 295 | 799 | No | joint Wald over 6 interaction terms |
| Total Bilirubin | 6. log, spline on day (joint test) | joint p=0.3993 | 295 | 799 | No | joint Wald over 3 interaction terms |
| Total Bilirubin | 7. log, linear, doubly robust | +0.008 (-0.012 to +0.029); p=0.4360 | 203 | 566 | No |  |
| Total Bilirubin | 8. log, adjusted for own day-1 value | +0.015 (-0.003 to +0.034); p=0.1087 | 234 | 674 | No |  |
| Total Bilirubin | 9. within-patient change from day 1 | Not estimable | 234 | 674 | — | Mixed model singular; see note below |
| Total Bilirubin | 10. log, + source database | +0.014 (-0.004 to +0.032); p=0.1192 | 295 | 799 | No |  |
| ALP | 1. published (raw, linear, RI) | +6.969 (+2.486 to +11.452); p=0.0023 | 295 | 797 | Yes |  |
| ALP | 2. log(x+1), linear, RI | +0.027 (+0.002 to +0.052); p=0.0358 | 295 | 797 | Yes |  |
| ALP | 3. raw, linear, random slope | +3.326 (-3.819 to +10.470); p=0.3616 | 295 | 797 | No |  |
| ALP | 4. log, linear, random slope | +0.006 (-0.031 to +0.043); p=0.7450 | 295 | 797 | No |  |
| ALP | 5. log, day as factor (joint test) | joint p=0.3413 | 295 | 797 | No | joint Wald over 6 interaction terms |
| ALP | 6. log, spline on day (joint test) | joint p=0.0990 | 295 | 797 | No | joint Wald over 3 interaction terms |
| ALP | 7. log, linear, doubly robust | +0.041 (+0.010 to +0.072); p=0.0090 | 204 | 570 | Yes |  |
| ALP | 8. log, adjusted for own day-1 value | +0.038 (+0.012 to +0.064); p=0.0041 | 234 | 672 | Yes |  |
| ALP | 9. within-patient change from day 1 | Not estimable | 234 | 672 | — | Mixed model singular; see note below |
| ALP | 10. log, + source database | +0.026 (+0.002 to +0.051); p=0.0374 | 295 | 797 | Yes |  |

*Specification 1 is on the raw scale (mg/dL/day, U/L/day); all others on log(x+1). RI, random intercept. Specifications 7–8 use fewer patients because they require complete day-1 covariates. Specification 9 (within-patient change from day 1 as a mixed model) returned a singular covariance matrix; specification 8 (adjustment for each patient's own day-1 value) addresses the same question. Bilirubin: null under every specification (p 0.109–0.436). ALP: significant under the primary, log-linear, doubly robust, day-1-adjusted and source-adjusted specifications; not under random-slope or flexible-day specifications.*

**Figure S5.** Group × day interaction under the model specifications with a single point estimate: raw scale (units per day) and log(x+1) scale. Point estimates with 95% confidence intervals for the group × day interaction. Orange: p<0.05; teal: p≥0.05. Dashed line: no difference in daily slope between arms. Specifications 5 and 6 (joint Wald tests) and 9 (not estimable) are reported in Table S5 only.

**Table S5b.** All trajectory outcomes with Benjamini–Hochberg correction across the full family, log(x+1) scale.

| **Outcome** | **Group × day (95% CI); p** | **q (full family)** | **Significant after correction** | **Patients** | **Observations** |
| --- | --- | --- | --- | --- | --- |
| Total Bilirubin | +0.014 (-0.003 to +0.032); p=0.1123 | 0.1605 | No | 295 | 799 |
| AST | +0.061 (+0.002 to +0.121); p=0.0427 | 0.0947 | No | 295 | 798 |
| ALT | +0.050 (+0.001 to +0.098); p=0.0474 | 0.0947 | No | 293 | 791 |
| ALP | +0.027 (+0.002 to +0.052); p=0.0358 | 0.0947 | No | 295 | 797 |
| INR | Model did not converge | — | — | 270 | 755 |
| Platelets | +0.433 (-2.147 to +3.014); p=0.7421 | 0.7421 | No | 363 | 1817 |
| Lactate | +0.294 (+0.044 to +0.544); p=0.0211 | 0.0947 | No | 273 | 509 |
| Albumin | -0.015 (-0.043 to +0.014); p=0.3230 | 0.4038 | No | 286 | 716 |
| MELD-Na | +0.966 (+0.323 to +1.609); p=0.0032 | 0.0323 | Yes | 218 | 430 |
| Hepatic SOFA Score | -0.009 (-0.044 to +0.026); p=0.6315 | 0.7016 | No | 295 | 799 |
| FIB-4 Index | +0.515 (-0.080 to +1.110); p=0.0897 | 0.1495 | No | 291 | 770 |

*Confirmatory family (Methods 2.3): total bilirubin and ALP. This table widens correction to all tested trajectories in response to Reviewer 2 Major 8. Within the two-analyte confirmatory family, ALP is significant (q = 0.005) and bilirubin is not. Under full-family correction, AST, ALT, ALP and lactate each have raw p<0.05 but q = 0.095, and bilirubin remains null. MELD-Na alone survives (q = 0.032); it is a composite driven by creatinine and INR and is interpreted as a severity descriptor (Discussion). The INR model did not converge.*

**Table S6.** Patients contributing a value on each study day, by analyte and arm.

| **Analyte** | **Arm** | **Day 1** | **Day 2** | **Day 3** | **Day 4** | **Day 5** | **Day 6** | **Day 7** | **Missing, day 7** |
| --- | --- | --- | --- | --- | --- | --- | --- | --- | --- |
| Total Bilirubin | DPP-4i (n=105) | 60 | 35 | 24 | 25 | 22 | 13 | 11 | 89.5% |
| Total Bilirubin | Metformin (n=297) | 174 | 116 | 97 | 76 | 64 | 46 | 36 | 87.9% |
| AST | DPP-4i (n=105) | 60 | 36 | 26 | 26 | 21 | 13 | 10 | 90.5% |
| AST | Metformin (n=297) | 171 | 114 | 96 | 75 | 64 | 49 | 37 | 87.5% |
| ALT | DPP-4i (n=105) | 60 | 36 | 26 | 26 | 21 | 13 | 11 | 89.5% |
| ALT | Metformin (n=297) | 172 | 114 | 94 | 74 | 63 | 47 | 34 | 88.6% |
| ALP | DPP-4i (n=105) | 60 | 35 | 25 | 26 | 21 | 13 | 11 | 89.5% |
| ALP | Metformin (n=297) | 174 | 114 | 95 | 75 | 63 | 48 | 37 | 87.5% |
| INR | DPP-4i (n=105) | 58 | 32 | 25 | 22 | 18 | 20 | 14 | 86.7% |
| INR | Metformin (n=297) | 166 | 103 | 71 | 70 | 58 | 50 | 48 | 83.8% |
| Platelets | DPP-4i (n=105) | 90 | 83 | 76 | 63 | 54 | 48 | 40 | 61.9% |
| Platelets | Metformin (n=297) | 256 | 245 | 233 | 199 | 164 | 146 | 120 | 59.6% |
| Lactate | DPP-4i (n=105) | 52 | 21 | 16 | 11 | 7 | 7 | 4 | 96.2% |
| Lactate | Metformin (n=297) | 181 | 81 | 43 | 27 | 20 | 21 | 18 | 93.9% |
| Albumin | DPP-4i (n=105) | 55 | 34 | 23 | 20 | 21 | 17 | 11 | 89.5% |
| Albumin | Metformin (n=297) | 158 | 105 | 77 | 67 | 53 | 45 | 30 | 89.9% |
| MELD-Na | DPP-4i (n=105) | 43 | 18 | 10 | 13 | 7 | 4 | 4 | 96.2% |
| MELD-Na | Metformin (n=297) | 129 | 64 | 35 | 36 | 26 | 21 | 20 | 93.3% |
| Hepatic SOFA Score | DPP-4i (n=105) | 60 | 35 | 24 | 25 | 22 | 13 | 11 | 89.5% |
| Hepatic SOFA Score | Metformin (n=297) | 174 | 116 | 97 | 76 | 64 | 46 | 36 | 87.9% |
| FIB-4 Index | DPP-4i (n=105) | 60 | 33 | 26 | 24 | 20 | 11 | 9 | 91.4% |
| FIB-4 Index | Metformin (n=297) | 168 | 112 | 93 | 73 | 62 | 45 | 34 | 88.6% |

*Days indexed from time zero. One measurement per patient-day. Attrition is similar in the two arms for every analyte. Percentage missing on every day is given in the workbook (sheet S6).*

**Figure S6.** Proportion of each arm contributing a measurement on days 1–7, by analyte. Attrition curves are near-parallel between arms, indicating that differential observation does not explain between-arm differences in slope.

**Figure S9.** Daily trajectories of albumin, INR, aPTT, platelets, lactate, MELD-Na, hepatic SOFA subscore and FIB-4 over study days 1–7, by exposure arm. Lactate and FIB-4 are geometric means (back-transformed mean of log[x+1]); all others are arithmetic means; 95% confidence bands. Numbers beneath each panel are patients contributing a value on that day; days with fewer than three contributing patients in an arm are not plotted. Fibrinogen is not shown because fewer than seven DPP-4 inhibitor patients contributed a value on any day.

**Table S6b.** Observation model: predictors of contributing a measurement on a given day.

| **Analyte** | **OR, exposed arm** | **p** | **OR per study day** | **OR per SOFA point** | **Patient-days** |
| --- | --- | --- | --- | --- | --- |
| Total Bilirubin | 1.47 | 1.000 | 3.77 | 2.28 | 799 |
| ALP | 1.48 | 1.000 | 3.75 | 2.29 | 797 |

*Fitted values from this model supply the inverse-probability-of-observation weights in Table S6c. The p-value of 1.000 for the exposure term reflects a non-informative Wald statistic in the weight model and should not be read as evidence of equal observation probability.*

**Table S6c.** Informative-observation sensitivity analyses, log(x+1) scale.

| **Analyte** | **Analysis** | **Group × day (95% CI); p** | **Patients** | **Observations** | **p < 0.05** |
| --- | --- | --- | --- | --- | --- |
| Total Bilirubin | IPOW-weighted GEE (exchangeable) | +0.020 (-0.012 to +0.051); p=0.2294 | 295 | 799 | No |
| Total Bilirubin | restricted to >=3 measurements (237 patients dropped) | +0.017 (-0.002 to +0.036); p=0.0859 | 127 | 567 | No |
| Total Bilirubin | survivors to discharge only | +0.013 (-0.007 to +0.033); p=0.2073 | 237 | 645 | No |
| Total Bilirubin | days 1-4 only (intact denominator) | +0.002 (-0.030 to +0.033); p=0.9251 | 288 | 607 | No |
| ALP | IPOW-weighted GEE (exchangeable) | +0.035 (-0.015 to +0.085); p=0.1662 | 295 | 797 | No |
| ALP | restricted to >=3 measurements (238 patients dropped) | +0.038 (+0.011 to +0.066); p=0.0060 | 126 | 563 | Yes |
| ALP | survivors to discharge only | +0.004 (-0.023 to +0.031); p=0.7690 | 237 | 643 | No |
| ALP | days 1-4 only (intact denominator) | +0.016 (-0.030 to +0.062); p=0.4905 | 289 | 604 | No |

*The IPOW point estimate for ALP (+0.035) is larger than the unweighted estimate (+0.027) but less precise. Restriction to patients with at least three measurements retains significance. The estimate is not significant among survivors to discharge or when follow-up is truncated at day 4, consistent with the linear-day dependence documented in Table S5. The bilirubin null holds in every analysis.*

**Figure S6c.** Informative-observation sensitivity analyses, log(x+1) scale. Point estimates with 95% confidence intervals for the group × day interaction. Orange: p<0.05; teal: p≥0.05. Dashed line: no difference in daily slope between arms.

# **Section 7. Mortality**

*Addresses Reviewer 2 Major 10 and Reviewer 3 Point 2.*

**Table S7a.** Mortality by endpoint, with risk ratios, confidence intervals and E-values.

| **Endpoint** | **DPP-4i, n/N (%)** | **Metformin, n/N (%)** | **Risk difference (pp)** | **RR (95% CI)** | **Fisher p** | **E-value, point** | **E-value, CI** |
| --- | --- | --- | --- | --- | --- | --- | --- |
| In-hospital (all three databases) | 22/105 (21.0) | 40/297 (13.5) | 7.5 | 1.56 (0.97 to 2.49) | 0.083 | 2.49 | 1.00 |
| In-hospital (MIMIC-IV only) | 11/35 (31.4) | 14/105 (13.3) | 18.1 | 2.36 (1.18 to 4.70) | 0.022 | 4.15 | 1.64 |
| 30-day (MIMIC-IV only) | 9/35 (25.7) | 17/105 (16.2) | 9.5 | 1.59 (0.78 to 3.24) | 0.218 | 2.56 | 1.00 |
| 90-day (MIMIC-IV only) | 11/35 (31.4) | 27/105 (25.7) | 5.7 | 1.22 (0.68 to 2.20) | 0.517 | 1.74 | 1.00 |

*Linked out-of-hospital death dates are available only in MIMIC-IV; eICU-CRD records none, and the completeness of the few post-discharge death dates in NWICU cannot be verified. Thirty- and 90-day mortality are therefore restricted to MIMIC-IV (35 exposed, 105 comparators); the in-hospital MIMIC-IV row is shown for comparison. A previous version reported 30- and 90-day mortality for MIMIC-IV and NWICU combined (46 exposed, 132 comparators: risk ratios 1.43 and 1.13); the corrected MIMIC-IV-only estimates do not change the conclusions. All mortality analyses are exploratory.*

**Figure S7a.** Risk ratios for mortality, DPP-4 inhibitor versus metformin, with 95% confidence intervals (log scale): in-hospital mortality in all three databases; in-hospital mortality restricted to documented pre-admission exposure and the exposed patients' own matched comparators (Table S7e, row 2); and 30- and 90-day mortality in MIMIC-IV, the only database with linked out-of-hospital death dates. All mortality analyses are exploratory.

**Table S7b.** In-hospital mortality accounting for the matched design.

| **Model** | **Effect measure** | **Estimate (95% CI)** | **p** | **Informative matched sets** | **Adjustment** |
| --- | --- | --- | --- | --- | --- |
| Conditional logistic, stratified on matched set | OR | 1.74 (0.96 to 3.17) | 0.068 | 47 | None |
| Poisson GEE, clustered on matched set | RR | 1.55 (0.98 to 2.47) | 0.063 | 105 | None |
| Poisson GEE + adjustment (20 covariates) | RR | Not estimable (complete separation) | — | 24 | 20 covariates (see note) |

*Both matched-design analyses agree with the unadjusted risk ratio (1.56). The model adjusting for all 20 residually imbalanced covariates (albumin, sulfonylurea, fibrinogen, sodium, MELD-Na, thiazolidinedione, aPTT, bilirubin, meglitinide, AST, chronic liver disease, hepatic SOFA, SGLT2 inhibitor, heart failure, INR, prandial insulin, ALP, SOFA, age, eGFR) against 62 events produced complete separation and returned no usable estimate; it is reported for transparency and not interpreted.*

**Table S7c.** Mortality ascertainment by source database.

| **Database** | **Matched, n** | **In-hospital death recorded, n** | **Missing (%)** | **30-day status available, n** | **90-day status available, n** | **Post-discharge vital status** |
| --- | --- | --- | --- | --- | --- | --- |
| eICU-CRD | 224 | 224 | 0.0 | 0 | 0 | no |
| MIMIC-IV | 140 | 140 | 0.0 | 140 | 140 | yes |
| NWICU | 38 | 38 | 0.0 | 38 | 38 | no |

*In-hospital mortality is complete and equivalently ascertained in all three databases. MIMIC-IV links out-of-hospital death dates; NWICU carries a small number of post-discharge death dates whose completeness cannot be verified, so 30- and 90-day mortality are analysed in MIMIC-IV only (Table S7a).*

**Table S7e.** In-hospital mortality restricted to documented pre-admission exposure.

| **Analysis** | **DPP-4i deaths/N (%)** | **Metformin deaths/N (%)** | **Risk difference (pp)** | **RR (95% CI)** | **Fisher p** | **E-value (point / CI)** |
| --- | --- | --- | --- | --- | --- | --- |
| Reference: full matched cohort (all recorded exposure) | 22/105 (21.0) | 40/297 (13.5) | 7.5 | 1.56 (0.97 to 2.49) | 0.083 | 2.49 / 1.00 |
| 1. Pre-admission exposed vs all comparators | 12/63 (19.0) | 40/297 (13.5) | 5.6 | 1.41 (0.79 to 2.54) | 0.243 | 2.18 / 1.00 |
| 2. Pre-admission exposed vs own matched comparators (63 matched sets) | 12/63 (19.0) | 27/175 (15.4) | 3.6 | 1.23 (0.67 to 2.29) | 0.553 | 1.77 / 1.00 |
| 2b. Conditional logistic on matched sets (29 informative sets) | — | — | — | OR 1.26 (0.58 to 2.72) | 0.565 | — |
| 3. In-admission exposed vs all comparators | 10/42 (23.8) | 40/297 (13.5) | 10.3 | 1.77 (0.96 to 3.26) | 0.101 | 2.93 / 1.00 |
| 4. eICU-CRD: pre-admission exposed vs eICU-CRD comparators | 11/59 (18.6) | 26/165 (15.8) | 2.9 | 1.18 (0.62 to 2.24) | 0.683 | 1.65 / 1.00 |
| 4. MIMIC-IV: pre-admission exposed vs MIMIC-IV comparators | 1/2 (50.0) | 14/105 (13.3) | 36.7 | not estimable (zero events or <5 exposed) | 0.262 | — |
| 4. MIMIC-IV: in-admission exposed vs MIMIC-IV comparators | 10/33 (30.3) | 14/105 (13.3) | 17.0 | 2.27 (1.12 to 4.63) | 0.035 | 3.97 / 1.48 |
| 4. NWICU: pre-admission exposed vs NWICU comparators | 0/2 (0.0) | 0/27 (0.0) | 0.0 | not estimable (zero events or <5 exposed) | 1.000 | — |
| 4. NWICU: in-admission exposed vs NWICU comparators | 0/9 (0.0) | 0/27 (0.0) | 0.0 | not estimable (zero events or <5 exposed) | 1.000 | — |

*Pre-admission exposure as defined in Table S2b (63 of 105 exposed patients). Row 2, which compares pre-admission exposed patients with their own matched comparators and so preserves the matched-set structure, is the primary restricted analysis; row 2b is the corresponding conditional logistic regression (29 informative sets). Row 3 shows the 42 patients whose exposure was first recorded during the admission. Rows with zero events or fewer than five exposed patients are not estimated. All mortality analyses are exploratory and non-causal.*

# **Section 8. Full trajectory model output, specification summaries, peak/nadir outcomes, minimum detectable effects and E-values**

*Addresses Reviewer 2 Majors 7 and 9 and Reviewer 3 Points 1 and 2.*

**Table S8.** Primary raw-scale mixed-model output for every trajectory analyte (the full version of Table 2).

| **Analyte** | **Group effect (95% CI); p** | **Metformin daily slope (95% CI); p** | **Group × day interaction (95% CI); p** | **q (confirmatory family)** |
| --- | --- | --- | --- | --- |
| Total Bilirubin | -0.280 (-0.656, 0.096); p=0.144 | -0.055 (-0.092, -0.017); p=0.004 | 0.048 (-0.029, 0.126); p=0.221 | 0.221 |
| ALP | -6.981 (-30.912, 16.951); p=0.568 | -0.682 (-2.840, 1.475); p=0.535 | 6.969 (2.486, 11.452); p=0.002 | 0.005 |
| Platelets | -5.053 (-32.052, 21.946); p=0.714 | 3.585 (2.295, 4.875); p<0.001 | 0.433 (-2.147, 3.014); p=0.742 | — |
| Lactate | -0.321 (-1.098, 0.457); p=0.419 | -0.291 (-0.409, -0.172); p<0.001 | 0.294 (0.044, 0.544); p=0.021 | — |
| Albumin | 0.146 (-0.024, 0.316); p=0.093 | -0.048 (-0.063, -0.034); p<0.001 | -0.015 (-0.043, 0.014); p=0.323 | — |
| MELD-Na | -1.915 (-4.484, 0.654); p=0.144 | -0.464 (-0.734, -0.195); p<0.001 | 0.966 (0.323, 1.609); p=0.003 | — |
| Hepatic SOFA Score | -0.045 (-0.237, 0.148); p=0.647 | 0.002 (-0.015, 0.019); p=0.837 | -0.009 (-0.044, 0.026); p=0.631 | — |
| FIB-4 Index | -0.369 (-2.906, 2.168); p=0.776 | -0.276 (-0.559, 0.007); p=0.056 | 0.515 (-0.080, 1.110); p=0.090 | — |
| AST | 49.473 (-47.463, 146.409); p=0.317 | -10.651 (-22.970, 1.669); p=0.090 | 7.011 (-18.507, 32.529); p=0.590 | — |
| ALT | -14.530 (-94.684, 65.625); p=0.722 | -3.303 (-13.261, 6.654); p=0.516 | 17.273 (-2.948, 37.493); p=0.094 | — |
| INR | -0.045 (-0.321, 0.231); p=0.750 | -0.028 (-0.061, 0.006); p=0.102 | 0.001 (-0.065, 0.068); p=0.970 | — |

*Model: value ~ group × day with a random intercept per patient, metformin as reference, raw units (mg/dL, U/L, g/dL, mmol/L, ×10⁹/L or points). q is reported only for the two-analyte confirmatory family (total bilirubin, ALP). AST, ALT and INR were fitted on values divided by their standard deviation for numerical stability, with coefficients converted back to raw units, using the Powell optimiser after the default optimiser returned a singular matrix. A patient-clustered OLS check gave concordant interaction estimates: AST +0.73 U/L/day (95% CI −46.9 to 48.3; p=0.976), ALT +11.5 (−26.5 to 49.6; p=0.552), INR −0.002 (−0.077 to 0.073; p=0.961).*

**Table S8a.** Every ALP estimate produced for the revision.

| **Source table** | **Analysis** | **Group × day (95% CI); p** | **Patients** | **p < 0.05** |
| --- | --- | --- | --- | --- |
| S5 | 1. published (raw, linear, RI) | +6.969 (+2.486 to +11.452); p=0.0023 | 295 | Yes |
| S5 | 2. log(x+1), linear, RI | +0.027 (+0.002 to +0.052); p=0.0358 | 295 | Yes |
| S5 | 3. raw, linear, random slope | +3.326 (-3.819 to +10.470); p=0.3616 | 295 | No |
| S5 | 4. log, linear, random slope | +0.006 (-0.031 to +0.043); p=0.7450 | 295 | No |
| S5 | 5. log, day as factor (joint test) | joint p=0.3413 | 295 | No |
| S5 | 6. log, spline on day (joint test) | joint p=0.0990 | 295 | No |
| S5 | 7. log, linear, doubly robust | +0.041 (+0.010 to +0.072); p=0.0090 | 204 | Yes |
| S5 | 8. log, adjusted for own day-1 value | +0.038 (+0.012 to +0.064); p=0.0041 | 234 | Yes |
| S5 | 10. log, + source database | +0.026 (+0.002 to +0.051); p=0.0374 | 295 | Yes |
| S4 | published (age, bmi, male, SOFA, eGFR) | +0.027 (+0.002 to +0.052); p=0.0358 | 295 | Yes |
| S4 | eGFR omitted (age, bmi, male, SOFA) | +0.007 (-0.021 to +0.034); p=0.6303 | 294 | No |
| S4 | eGFR and SOFA omitted (age, bmi, male) | +0.007 (-0.019 to +0.032); p=0.6116 | 285 | No |
| S4b | A. all recorded exposure (published) | +0.027 (+0.002 to +0.052); p=0.0358 | 295 | Yes |
| S4b | B. exposure at/before time zero (61 exposed, 270 comparators) | +0.022 (-0.006 to +0.050); p=0.1232 | 270 | No |
| S4b | C. DPP-4i+metformin (n=45) vs metformin alone | +0.034 (-0.004 to +0.071); p=0.0827 | 250 | No |
| S4b | D. DPP-4i without metformin (n=60) vs metformin alone | +0.024 (-0.005 to +0.052); p=0.1084 | 261 | No |
| S4b | E. adjusted for concomitant therapy (ad_sulfonylurea, ad_basal_insulin, ad_prandial_insulin) | +0.027 (+0.002 to +0.052); p=0.0354 | 295 | Yes |
| S4b | F. excluding coded chronic liver disease (n=20 removed) | +0.033 (+0.006 to +0.060); p=0.0164 | 277 | Yes |
| S6c | IPOW-weighted GEE (exchangeable) | +0.035 (-0.015 to +0.085); p=0.1662 | 295 | No |
| S6c | restricted to >=3 measurements (238 patients dropped) | +0.038 (+0.011 to +0.066); p=0.0060 | 126 | Yes |
| S6c | survivors to discharge only | +0.004 (-0.023 to +0.031); p=0.7690 | 237 | No |
| S6c | days 1-4 only (intact denominator) | +0.016 (-0.030 to +0.062); p=0.4905 | 289 | No |
| S3d | all databases | +0.027 (+0.002 to +0.052); p=0.0358 | 295 | Yes |
| S3d | excluding eICU-CRD | +0.043 (-0.001 to +0.088); p=0.0541 | 105 | No |
| S3d | excluding MIMIC-IV | +0.015 (-0.015 to +0.046); p=0.3284 | 190 | No |
| S3d | excluding NWICU | +0.027 (+0.002 to +0.052); p=0.0358 | 295 | Yes |
| S3b | Database-specific: eICU-CRD | +0.015 (-0.015 to +0.046); p=0.3284 | 190 | No |
| S3b | Database-specific: MIMIC-IV | +0.043 (-0.001 to +0.088); p=0.0541 | 105 | No |
| S3b | Database-specific: NWICU | Did not converge | — | — |

*Of the 28 estimable rows, 12 retain p<0.05; the published specification recurs as the reference row in several tables. The signal is retained across scale, day-1 adjustment, doubly robust adjustment, source adjustment, concomitant-therapy adjustment, exclusion of chronic liver disease and restriction to patients with at least three measurements. It is not retained under random slopes, flexible day terms, removal of eGFR from the propensity model, restriction to exposure at or before time zero, IPOW, survivors only, truncation at day 4, or in either single database. It is reported as hypothesis-generating (main text Section 3.2).*

**Table S8b.** Every total-bilirubin (primary outcome) estimate produced for the revision.

| **Source table** | **Analysis** | **Group × day (95% CI); p** | **Patients** | **p < 0.05** |
| --- | --- | --- | --- | --- |
| S5 | 1. published (raw, linear, RI) | +0.048 (-0.029 to +0.126); p=0.2208 | 295 | No |
| S5 | 2. log(x+1), linear, RI | +0.014 (-0.003 to +0.032); p=0.1123 | 295 | No |
| S5 | 3. raw, linear, random slope | +0.050 (-0.051 to +0.151); p=0.3310 | 295 | No |
| S5 | 4. log, linear, random slope | +0.019 (-0.007 to +0.044); p=0.1556 | 295 | No |
| S5 | 5. log, day as factor (joint test) | joint p=0.4036 | 295 | No |
| S5 | 6. log, spline on day (joint test) | joint p=0.3993 | 295 | No |
| S5 | 7. log, linear, doubly robust | +0.008 (-0.012 to +0.029); p=0.4360 | 203 | No |
| S5 | 8. log, adjusted for own day-1 value | +0.015 (-0.003 to +0.034); p=0.1087 | 234 | No |
| S5 | 10. log, + source database | +0.014 (-0.004 to +0.032); p=0.1192 | 295 | No |
| S4 | published (age, bmi, male, SOFA, eGFR) | +0.014 (-0.003 to +0.032); p=0.1123 | 295 | No |
| S4 | eGFR omitted (age, bmi, male, SOFA) | -0.004 (-0.023 to +0.015); p=0.6761 | 293 | No |
| S4 | eGFR and SOFA omitted (age, bmi, male) | +0.010 (-0.010 to +0.030); p=0.3262 | 288 | No |
| S4b | A. all recorded exposure (published) | +0.014 (-0.003 to +0.032); p=0.1123 | 295 | No |
| S4b | B. exposure at/before time zero (61 exposed, 270 comparators) | +0.015 (-0.005 to +0.036); p=0.1448 | 269 | No |
| S4b | C. DPP-4i+metformin (n=45) vs metformin alone | +0.022 (-0.007 to +0.050); p=0.1312 | 250 | No |
| S4b | D. DPP-4i without metformin (n=60) vs metformin alone | +0.010 (-0.012 to +0.031); p=0.3686 | 260 | No |
| S4b | E. adjusted for concomitant therapy (ad_sulfonylurea, ad_basal_insulin, ad_prandial_insulin) | +0.015 (-0.003 to +0.032); p=0.1043 | 295 | No |
| S4b | F. excluding coded chronic liver disease (n=20 removed) | +0.011 (-0.007 to +0.030); p=0.2332 | 277 | No |
| S6c | IPOW-weighted GEE (exchangeable) | +0.020 (-0.012 to +0.051); p=0.2294 | 295 | No |
| S6c | restricted to >=3 measurements (237 patients dropped) | +0.017 (-0.002 to +0.036); p=0.0859 | 127 | No |
| S6c | survivors to discharge only | +0.013 (-0.007 to +0.033); p=0.2073 | 237 | No |
| S6c | days 1-4 only (intact denominator) | +0.002 (-0.030 to +0.033); p=0.9251 | 288 | No |
| S3d | all databases | +0.014 (-0.003 to +0.032); p=0.1123 | 295 | No |
| S3d | excluding eICU-CRD | +0.019 (-0.017 to +0.056); p=0.2979 | 107 | No |
| S3d | excluding MIMIC-IV | +0.009 (-0.009 to +0.027); p=0.3152 | 188 | No |
| S3d | excluding NWICU | +0.014 (-0.003 to +0.032); p=0.1123 | 295 | No |

*Every one of the 26 bilirubin estimates is null. The lowest p-value across all analyses is 0.086. The primary finding of no divergence in bilirubin trajectory is robust to model specification, propensity-score specification, estimand, exposure window, informative observation and database.*

**Figure S8b.** Every total-bilirubin (primary outcome) estimate produced for the revision: raw-scale specifications (top) and all log(x+1)-scale specifications, propensity-score specifications, estimand and exposure-window analyses, informative-observation analyses and leave-one-database-out analyses (bottom). All 26 confidence intervals include zero.

**Table S9a.** Minimum detectable group × day effect at 80% power and α = 0.05, raw scale.

| **Analyte** | **Observed group × day** | **SE** | **MDE per day** | **MDE over 7 days** | **Observed / MDE** | **Patients** |
| --- | --- | --- | --- | --- | --- | --- |
| Total Bilirubin | 0.0484 | 0.0396 | 0.1108 | 0.776 | 0.44 | 295 |
| ALP | 6.9691 | 2.2874 | 6.4084 | 44.859 | 1.09 | 295 |
| Platelets | 0.4333 | 1.3167 | 3.6888 | 25.822 | 0.12 | 363 |
| Lactate | 0.2938 | 0.1274 | 0.3570 | 2.499 | 0.82 | 273 |
| Albumin | -0.0145 | 0.0147 | 0.0412 | 0.289 | 0.35 | 286 |
| MELD-Na | 0.9659 | 0.3280 | 0.9189 | 6.432 | 1.05 | 218 |
| Hepatic SOFA Score | -0.0086 | 0.0179 | 0.0500 | 0.350 | 0.17 | 295 |
| FIB-4 Index | 0.5151 | 0.3036 | 0.8505 | 5.954 | 0.61 | 291 |

*Units: bilirubin mg/dL, ALP U/L, platelets ×10⁹/L, lactate mmol/L, albumin g/dL; MELD-Na, hepatic SOFA and FIB-4 in points. The observed ALP effect exceeds its MDE (ratio 1.09); the observed bilirubin effect is 44% of its MDE. AST, ALT and INR were not included in the MDE run.*

**Table S9b.** Minimum detectable in-hospital mortality effect at 80% power and α = 0.05.

| **Comparator risk (%)** | **Observed exposed risk (%)** | **n exposed** | **n comparator** | **Minimum detectable exposed risk (%)** | **Minimum detectable risk difference (pp)** | **Minimum detectable RR** |
| --- | --- | --- | --- | --- | --- | --- |
| 13.5 | 21.0 | 105 | 297 | 25.8 | 12.3 | 1.91 |

*The observed 7.5-point difference is below the 12.3-point MDE.*

**Table S9c.** E-values for the reported positive findings.

| **Finding** | **Estimate** | **CI limit nearest null** | **E-value, point** | **E-value, CI limit** |
| --- | --- | --- | --- | --- |
| In-hospital mortality | RR 1.56 | 0.97 | 2.49 | 1.00 |
| ALP 7-day separation (approximate RR scale) | d=0.553 SD -> RR~1.65 | 1.20 | 2.70 | 1.68 |

*For ALP, the seven-day separation was converted to a standardised difference (d = 0.553) and then to the approximate risk-ratio scale (RR ≈ 1.65). An unmeasured confounder associated with both exposure and ALP trajectory by a risk ratio of 2.7 each, beyond measured covariates, would be required to explain the point estimate; 1.68 to move the confidence limit to the null. E-values for 30- and 90-day mortality are given in Table S7a.*

**Table S10.** Peak and nadir values over study days 1–7.

| **Outcome** | **Patients with a value (DPP-4i / metformin)** | **DPP-4i, median [IQR]** | **Metformin, median [IQR]** | **p** |
| --- | --- | --- | --- | --- |
| Peak AST | 80 / 215 | 38.8 [24.0-111.8] | 38.0 [23.0-82.0] | 0.504 |
| Peak ALT | 80 / 213 | 33.0 [20.0-68.2] | 34.0 [18.0-66.5] | 0.619 |
| Peak bilirubin | 80 / 215 | 0.6 [0.5-1.1] | 0.7 [0.4-1.1] | 0.712 |
| Peak ALP | 79 / 216 | 105.0 [72.5-153.5] | 97.0 [72.4-142.0] | 0.489 |
| Peak INR | 69 / 201 | 1.4 [1.2-1.9] | 1.4 [1.2-1.8] | 0.821 |
| Nadir platelets | 94 / 269 | 164.2 [115.8-213.5] | 164.0 [112.5-236.0] | 0.702 |

*Units: AST, ALT and ALP U/L; bilirubin mg/dL; platelets ×10⁹/L. Mann–Whitney U test. Each patient contributes the highest (or, for platelets, lowest) value recorded on days 1–7; patients without any value for the analyte are excluded.*
